# MST1 mediates doxorubicin-induced cardiomyopathy by SIRT3 downregulation

**DOI:** 10.1007/s00018-023-04877-7

**Published:** 2023-08-11

**Authors:** Leonardo Schirone, Daniele Vecchio, Valentina Valenti, Maurizio Forte, Michela Relucenti, Annalisa Angelini, Tania Zaglia, Sonia Schiavon, Luca D’Ambrosio, Gianmarco Sarto, Rosita Stanzione, Elisa Mangione, Selenia Miglietta, Anna Di Bona, Marny Fedrigo, Alessandra Ghigo, Francesco Versaci, Vincenzo Petrozza, Simona Marchitti, Speranza Rubattu, Massimo Volpe, Junichi Sadoshima, Luigi Frati, Giacomo Frati, Sebastiano Sciarretta

**Affiliations:** 1 Department of Medical and Surgical Sciences and Biotechnologies, Sapienza University of Rome, Latina, Italy; 2 Department of Cardiology, Santa Maria Goretti Hospital, Latina, Italy; 3 IRCCS Neuromed, Pozzilli, Italy; 4 Department of Anatomical, Sapienza University of Rome, Histological, Forensic Medicine and Orthopaedic Sciences, Rome, Italy; 5 Department of Cardiac-Thoracic-Vascular Sciences and Public Health, University of Padova Medical School, Padua, Italy; 6 Veneto Institute of Molecular Medicine, Padua, Italy; 7 Department of Biomedical Sciences, University of Padova, Padua, Italy; 8 ICOT, UOC Anatomia Patologica, Latina, Italy; 9 Department of Molecular Biotechnology and Health Sciences, Molecular Biotechnology Center, University of Torino, Turin, Italy; 10 Department of Clinical and Molecular Medicine, (Sapienza University of Rome, S. Andrea Hospital), Rome, Italy; 11 Department of Cell Biology and Molecular Medicine, Rutgers New Jersey Medical School, Cardiovascular Research Institute, Newark, NJ USA; 12 Istituto Pasteur - Fondazione Cenci Bolognetti, Rome, Italy; 13 IRCCS San Raffaele, Rome, Italy

**Keywords:** Heart failure, Anthracyclines, Cardiomyopathy, Cardioncology, Cardiotoxicity, Hippo pathway

## Abstract

Heart failure is a major side effect of doxorubicin (DOX) treatment in patients with cancer. However, the mechanisms underlying the development of DOX-induced heart failure need to be addressed. This study aims to test whether the serine/threonine kinase MST1, a major Hippo pathway component, contributes to the development of DOX-induced myocardial injury. C57BL/6J WT mice and mice with cardiomyocyte-specific dominant-negative MST1 (kinase-dead) overexpression received three weekly injections of DOX, reaching a final cumulative dose of 18 mg/kg. Echocardiographic, histological and biochemical analyses were performed six weeks after the first DOX administration. The effects of MST1 inhibition on DOX-induced cardiomyocyte injury were also tested in vitro. MST1 signaling was significantly activated in cardiomyocytes in response to DOX treatment in vitro and in vivo. Wild-type (WT) mice treated with DOX developed cardiac dysfunction and mitochondrial abnormalities. However, these detrimental effects were abolished in mice with cardiomyocyte-specific overexpression of dominant-negative MST1 (DN-MST1) or treated with XMU-MP-1, a specific MST1 inhibitor, indicating that MST1 inhibition attenuates DOX-induced cardiac dysfunction. DOX treatment led to a significant downregulation of cardiac levels of SIRT3, a deacetylase involved in mitochondrial protection, in WT mice, which was rescued by MST1 inhibition. Pharmacological inhibition of SIRT3 blunted the protective effects of MST1 inhibition, indicating that SIRT3 downregulation mediates the cytotoxic effects of MST1 activation in response to DOX treatment. Finally, we found a significant upregulation of MST1 and downregulation of SIRT3 levels in human myocardial tissue of cancer patients treated with DOX. In summary, MST1 contributes to DOX-induced cardiomyopathy through SIRT3 downregulation.

**Supplementary Information:**

The online version contains supplementary material available at 10.1007/s00018-023-04877-7.

## Introduction

Doxorubicin is a drug belonging to the class of anthracyclines, which is primarily used as a chemotherapeutic agent in several malignancies. However, its therapeutic use is associated with severe adverse effects that have been reported over the past decades [1]. Doxorubicin-induced cardiomyopathy is a potentially lethal condition that hardly benefits from available therapies and may manifest acutely or chronically [2]. Cardiotoxicity has emerged as a significant side effect of doxorubicin (DOX) treatment, affecting nearly 30% of patients within five years after chemotherapy [3]. Remarkably, heart failure is the first non-cancer cause of death in DOX-treated patients [3]. Unfortunately, the signaling mechanisms underlying DOX-induced cardiotoxicity remain largely unclear.

MST1 is a major Hippo pathway component, a transduction signaling cascade that negatively regulates cell survival and growth [4]. The importance of MST1 in the regulation of cardiac stress response was demonstrated in various works that highlighted its role in the development of myocardial injury during stress [5, 6]. Cardiac overexpression of MST1 results in dilated cardiomyopathy without compensatory ventricular myocyte hypertrophy. MST1 activation also contributes to cell death and tissue injury in response to myocardial ischemia/reperfusion injury and chronic myocardial infarction. On the other hand, MST1 activation in response to pressure overload appears to be in part physiological by restraining cardiomyocyte dedifferentiation and proliferation [7]. To date, the role of MST1 in the genesis of cardiac derangements induced by DOX is unknown.

Therefore, this study aimed at exploring for the first time the role of MST1 in the development of DOX-induced cardiomyopathy.

## Materials and methods

The expanded version of the methods section is reported in Online Resource 1.

### Animal models

C57BL/6J transgenic mice with cardiomyocyte-specific DN-MST1 overexpression driven by *α-myosin heavy chain* promoter were previously described ^11^. Littermate wild-type animals were used as genetic controls of DN-MST1 mice. 3–5 months aged male and female mice were used for the experiments and were randomly allocated to different treatment groups. No a priori exclusion criteria were set. Cardiac function analyses were blinded. Commercially-available C57BL/6J animals (Charles River) were used for pharmacological MST1 inhibition experiments.

4’-Br-Resveratrol was purchased by R&S Chemicals, Inc. (Kannapolis, NC 28081 USA). A concentration of 0.2 mM was used in vitro. Mice were injected i.p. three times every week for six weeks with a dose of 10 mg/kg of 4’-Br-Resveratrol suspended in saline solution. XMU-MP-1 (Sigma) 0.1 mg/ml was injected i.p. three times every week for six weeks at a 1 mg/kg dose. At the end of the treatments, mice were anaesthetized and euthanised by cervical dislocation to harvest the hearts for *post-mortem* analyses (see details in *supplemental material*).

### Antibodies

Antibodies used for immunoblots were purchased from the indicated companies: MST1 (BD-611052 and H00006789-M02), GAPDH (CS-2118), P-LATS (CS-8654), LATS (SC-398560), Histone H3 (CS-9715), cl-CAS3 (CS-9661), cl-CAS9 (CS-9509), Actin-β (SC-69879), SIRT3 (SC-365175 and CS-5490), Vinculin (sc-25336), H2B (CS-12364), P_ser14_-H2B(CS-6959), Tri-Methyl-Histone (Lys27) H3 (CS-9733), Cardiac Troponin T (Thermo MA5-12,960), COX IV (CS- 4850) [BD = Bio-Rad; CS = Cell Signaling; H = Invitrogen; SC = Santa Cruz]. Anti-mouse and anti-rabbit HRP-linked antibodies were purchased from Bio-Rad and Cell Signaling Technology. All antibodies were diluted in 5% non-fat dry milk powder in 0.05% Tween 20 Tris-buffered saline (TBST) or 3% BSA in TBST.

### Chromatin immunoprecipitation (ChIP)

ChIP was performed using a previously described protocol [8]. All the buffers used for chromatin immunoprecipitation were supplemented with protease inhibitors (Roche). In brief, 10 × 10^6^ H9C2 cells (ATCC^®^ CRL-1446™) were infected with ad-LacZ or ad-DN-MST1 for 48 h and then treated with DOX 1 µM for four hours. Afterward, these were fixed with 1% formaldehyde for 10 min at room temperature (RT) and then sonicated in ice-cold lysis buffer (1% SDS, 10 mM EDTA, 50 mM Tris–HCl, pH 8.1) to obtain chromatin fragments of ~ 300 bp in length. The sheared chromatin was diluted tenfold in ‘dilution buffer’ (1% Triton X-100, 2 mM EDTA, 150 mM NaCl, 20 mM Tris–HCl, pH 8.1) and precleared with protein A-Sepharose (GE-Healthcare). The 5% of the supernatant was collected as the ‘input’. Chromatin was incubated with 5 μg Tri-Methyl-Histone H3 antibody at Lys 27 (CS-9733) or 5 μg normal goat IgG antibodies (Millipore). The immunoprecipitated complexes were recovered with protein A-Sepharose and washed with ice-cold buffers with the following protocol: one wash in buffer 1 (0.1% SDS, 1% Triton X-100, 2 mM EDTA, 150 mM NaCl, 20 mM Tris–HCl, pH 8.1), four washes in buffer 2 (0.1% SDS, 1% Triton X-100, 2 mM EDTA, 500 mM NaCl, 20 mM Tris–HCl, pH 8.1), one wash in buffer 3 (250 mM LiCl, 1% NP-40, 1% sodium deoxycholate, 1 mM EDTA, 10 mM Tris–HCl, pH 8.1) and three washes in TE buffer (10 mM Tris–HCl, pH 7.5, 1 mM EDTA). The immunocomplexes were then reverse crosslinked, and the DNA was purified and amplified by PCR for 30 cycles, run on a 1.5% agarose gel. Images were acquired using a ChemiDoc (Bio-Rad) and quantified for band intensity using ImageJ.

Sirt3 promoter fw (agagctgggaacacaaatac) and rev (caagaacgcggttacctt) were used as primers to amplify the promoter of *sirt3* in the proximity of the transcription starting site (TSS).

### Human samples

We analysed sections from human subjects who died of non-cardiac causes (n = 3) and subjects who underwent doxorubicin treatment and developed cardiomyopathy (n = 3). Ventricular samples were acquired during routine *post-mortem* investigations for the patients who died of non-cardiac causes and during the post-transplant evaluation of the native heart from oncological patients treated with DOX. Then, the samples were archived in the anatomical collection of the Institute of Pathological Anatomy of the University of Padova. Samples were anonymous to the investigators and used by the directives of the national committee of Bioethics and Recommendation (2006) of the Committee of the Ministers of member states of the EU on the use of samples of human origin for research and conform to the principles outlined in the Declaration of Helsinki.

### Immunofluorescence analysis of the human myocardium.

Three µm thick formalin-fixed paraffin-embedded human heart sections underwent antigen retrieval and IF staining following the protocol described [9]. Sections were analysed at the confocal microscope (Leica TCS SP5), and MST1 and SIRT3 fluorescence intensity were analysed using the software FiJi.

### Statistical analyses

All data are presented as mean ± SEM. A two-sided t-test was used to compare two independent groups for continuous variables. A one-way ANOVA analysis followed by a Bonferroni post hoc test was used when more than two independent groups were compared. Survival was analysed with a logrank (Mantel-Cox) test. Non-normal distributed measurements were analysed with a Mann–Whitney test. All statistical analyses were conducted with Prism statistical software (GraphPad, La Jolla, CA).

## Results

### MST1 upregulation mediates cardiomyocyte cytotoxicity in response to DOX

**Fig. 1:**
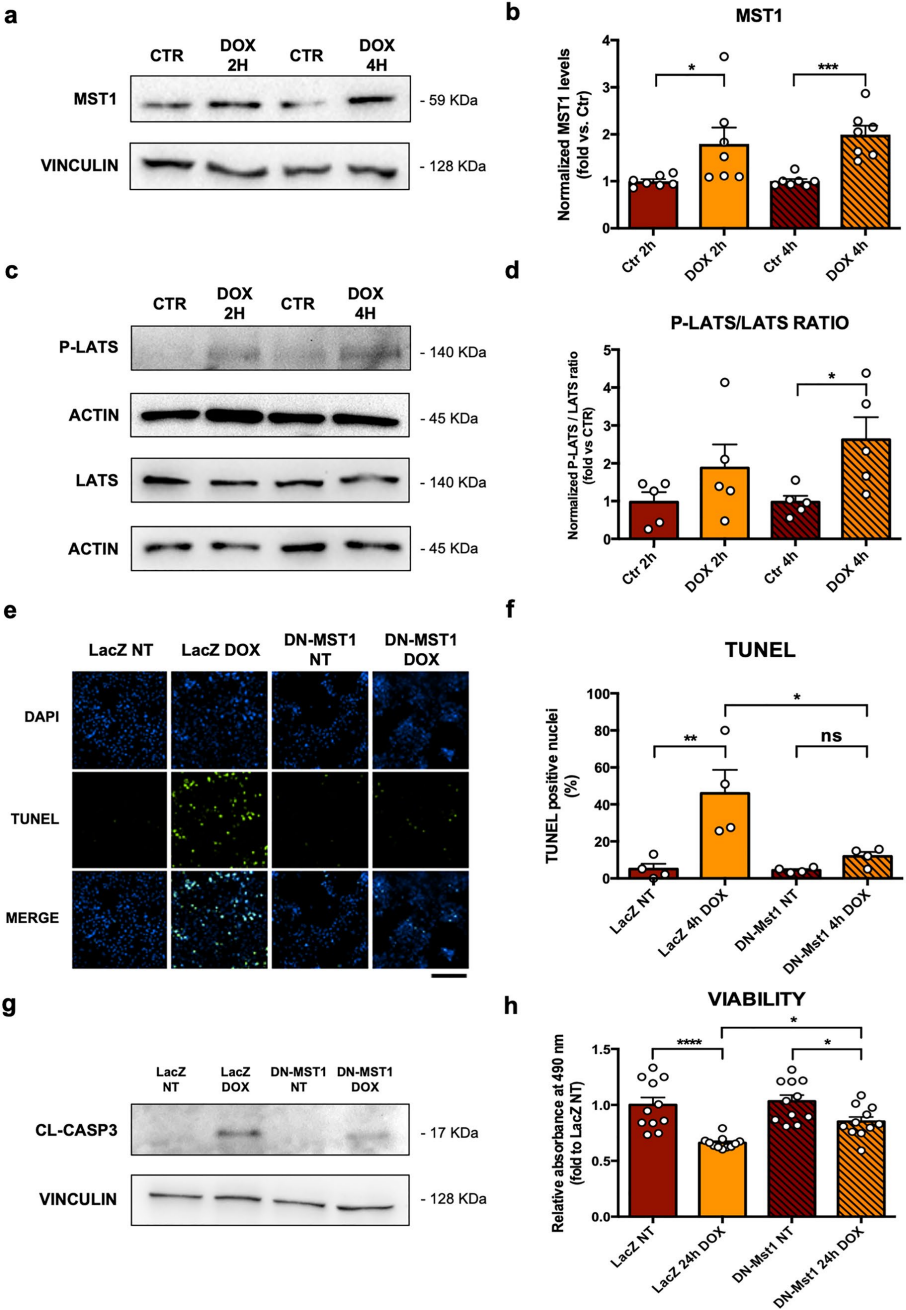
MST1 upregulation mediates cardiomyocyte cytotoxicity in response to DOX in vitro*,* **a**–**b** MST1 expression profile after two and four hours of treatment with 50 μM of doxorubicin in primary cardiomyocyte cultures. Densitometric data normalized by loading control represent mean ± SEM (*n* = 7 independent samples); **c**–**d** P-LATS and LATS expression profiles after two and four hours of treatment with 50 μM of doxorubicin. Densitometric data normalized by loading control and expressed as P-LATS/LATS ratio ± SEM (*n* = 5 independent samples); **e**–**f** TUNEL (*green)* staining of cardiomyocyte cultures infected with ad-LacZ or ad-DN-MST1 for 48 h, and then treated with doxorubicin (50 μM) for four hours. Nuclei were counterstained with DAPI. Data represent mean ± SEM (*n* = 4 independent samples). Scale bar = 200 μm; **g** Cl-Caspase 3 protein profile from cardiomyocyte cultures infected with ad-LacZ or ad-DN-MST1 for 48 h, then treated with doxorubicin (50 μM) for four hours. Representative images of five independent experiments; **h** MTS colorimetric assay of cardiomyocyte cultures infected with ad-LacZ or ad-DN-MST1 for 48 h and then treated with doxorubicin (50 μM) for 24 h. Data represent mean ± SEM (*n* = 11 independent samples). Data were analysed with a two-tailed Student’s t-test (b, d) or one-way ANOVA with Bonferroni post-hoc test (f–h). *P ≤ 0.05; **P ≤ 0.01; ***P ≤ 0.001; ****P ≤ *0.0001; ns* = *not significant (P* > *0.05)*

Neonatal rat ventricular cardiomyocytes (CMs) were treated with 50 µM of doxorubicin (DOX) for two and four hours. MST1 expression levels are significantly increased in CMs treated with DOX, as compared to control cells (Fig. 1a, b). Phosphorylation levels of LATS, a downstream target of MST1, are also significantly increased after four hours of DOX treatment (Fig. 1c, d). Overall, this data demonstrates that MST1 signaling is rapidly activated in response to DOX treatment in CMs in vitro. Then, we evaluated whether MST1 activation contributes to DOX-induced cytotoxicity. Cardiomyocytes (CMs) were infected with either an adenovirus overexpressing a ‘kinase-dead’ form of MST1 (K59R) with a dominant-negative effect (ad-DN-MST1) or a control adenovirus overexpressing β-Galactosidase (ad-LacZ) 48 h before DOX treatment. Effective MST1 inhibition was achieved, as indicated by reduced LATS phosphorylation in ad-DN-MST1-infected CM treated with DOX compared to cells with LacZ overexpression (Online Resource 2—Supplementary Fig. 1a-b). DOX treatment significantly increases apoptosis, as indicated by increased TUNEL-positive CMs. However, MST1 inhibition by DN-MST1 overexpression significantly reduces TUNEL-positive cells after DOX treatment, as compared to LacZ overexpression (Fig. 1e, f, Online Resource 2—Supplementary Fig. 1c). Furthermore, MST1 inhibition significantly attenuates the increase of cleaved-Caspase 3 levels induced by DOX treatment with respect to control CMs (Fig. 1g, Online Resource 2—Supplementary Fig. 1d). In addition, DOX treatment significantly reduces the viability of control CMs with LacZ overexpression with respect to untreated samples, as assessed by an MTS assay. In contrast, MST1 inhibition by DN-MST1 overexpression preserves CM viability in response to DOX treatment (Fig. 1h). Overall, these data demonstrate that upregulation of endogenous MST1 contributes to the cytotoxic effects of DOX treatment in CMs.

### MST1 activation contributes to DOX-induced mitochondrial oxidative stress and SIRT3 downregulation

**Fig. 2:**
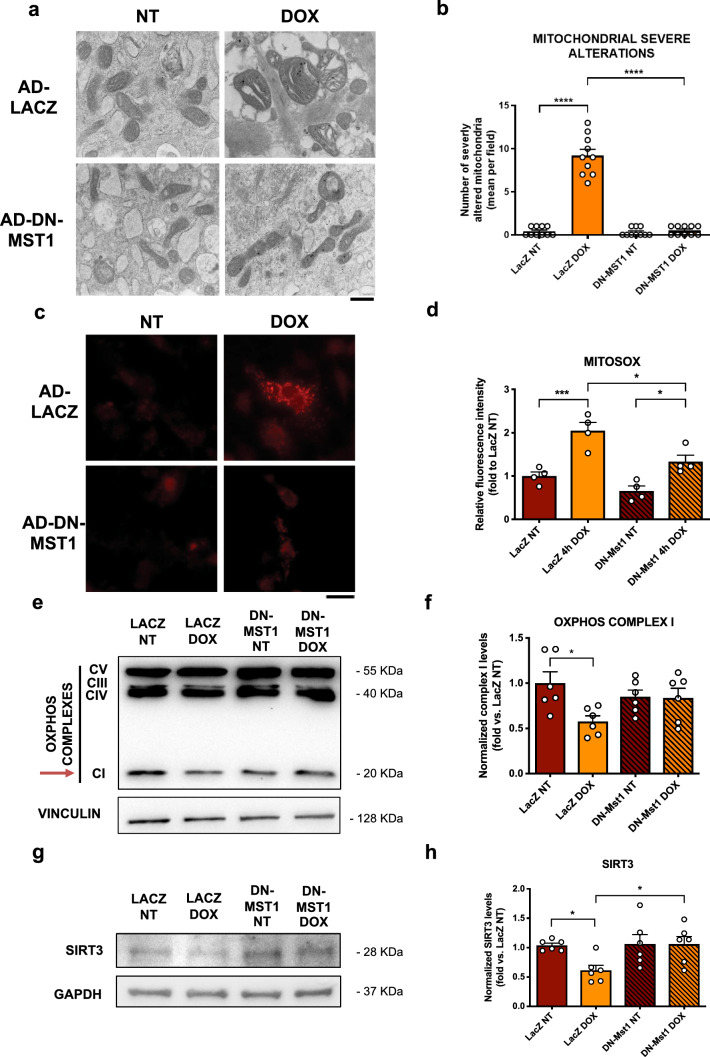
MST1 inhibition attenuates DOX-induced mitochondrial oxidative stress and preserves SIRT3 expression levels, **a** Representative TEM images of myocardial mitochondria from cardiomyocyte cultures infected with ad-LacZ or ad-DN-MST1 for 48 h and then treated with doxorubicin (50 μM) for four hours. Scale bar = 0.4 μm; **b** quantification of the number of mitochondria presenting severe alterations per microscopic field*.* Data represent mean ± SEM (*n* = 10 microscopic fields from 5 independent samples); **c**–**d** MitoSOX staining of cardiomyocyte cultures infected with ad-LacZ or ad-DN-MST1 for 48 h, and then treated with doxorubicin (50 μM) for four hours. Data represent mean ± SEM (*n* = 4 independent samples). Scale bar = 25 μm; (**e**–**f**) OXPHOS complexes expression profile and complex I quantification from cardiomyocyte cultures infected with ad-LacZ or ad-DN-MST1 for 48 h, then treated with doxorubicin (50 μM) for four hours. Densitometric data normalized by loading control represent mean ± SEM (*n* = 6 independent samples); **g**–**h** SIRT3 expression profile in cardiomyocytes infected with ad-LacZ or ad-DN-MST1 for 48 h, and then treated with doxorubicin (50 μM) for four hours. Densitometric data normalized by loading control represent mean ± SEM (*n* = 6 independent samples). Data were analysed with one-way ANOVA with Bonferroni post-hoc test. *P ≤ 0.05; ***P ≤ 0.001; ****P ≤ 0.0001

Previous work demonstrated that DOX induces mitochondrial damage and mitochondrial-derived oxidative stress by the interaction of a metabolized semiquinone form of DOX with cardiolipin at the mitochondrial membrane, thereby producing high levels of reactive oxygen species (ROS) [10]. Similarly, previous work demonstrated that MST1 contributes to mitochondria-dependent apoptosis in response to oxidative stress in CMs [11]. Therefore, we hypothesized that MST1 inhibition might be beneficial in response to DOX treatment by reducing mitochondrial damage. CMs were infected with ad-LACZ or ad-DN-MST1 for 48 h and then treated for four hours with DOX. Transmission electronic microscopy (TEM) observations at baseline and in response to four hours of DOX treatment showed that DOX significantly induces mitochondrial structural damage and swelling. Conversely, MST1 inhibition by DN-MST1 overexpression preserves mitochondrial morphology after DOX treatment (Fig. 2a, b, Online Resource 2—Supplementary Fig. 2a). No significant changes were observed in their numerosity (Online Resource 2—Supplementary Fig. 2b). Similarly, mitochondrial oxidative stress was assessed by using mitoSOX™ and mitoTracker Green™ fluorescent dyes, and we found that DOX treatment significantly increases mitochondrial ROS with respect to untreated CMs. In contrast, mitochondrial ROS production is attenuated considerably in DOX-treated CMs with DN-MST1 overexpression, indicating that MST1 activation in response to DOX treatment contributes to mitochondrial oxidative stress (Fig. 2c, d, Online Resource 2—Supplementary Fig. 2c). Lastly, we observed a reduced mitochondrial complex I (OXPHOS CI) assembly in primary CMs treated with DOX and ad-LacZ. Differently, samples transduced with ad-DN-MST1 show preserved levels of intact OXPHOS CI (Fig. 2e, f). No significant changes were found in complexes III, IV and V (Online Resource 2—Supplementary Fig. 2d-e).

Previous studies showed that DOX treatment promotes cardiotoxicity through the downregulation of SIRT3, a mitochondrial deacetylase that preserves a variety of essential mitochondrial functions, including ROS scavenging, mitochondrial pore opening and electron transport chain oxidative function [12]. MST1 was previously found to inhibit SIRT3 in response to cellular stress [13]. Therefore, we assessed the levels of SIRT3 in CMs with ad-LACZ, ad-MST1 or ad-DN-MST1 overexpression with or without four hours of DOX treatment. We found a significant downregulation of SIRT3 protein levels in LACZ-overexpressing CMs in response to DOX treatment. In contrast, SIRT3 levels are preserved in CMs transduced with ad-DN-MST1 (Fig. 2g, h), suggesting that MST1 contributes to DOX-induced SIRT3 downregulation in CMs. We then checked whether the preservation of SIRT3 levels mediates the beneficial effects of MST1 inhibition on CM survival and apoptosis in response to DOX treatment. For this purpose, we checked whether the administration of 4’-Br-Resveratrol, a novel pharmacological competitive inhibitor of SIRT3, suppresses the beneficial effects of DN-MST1 overexpression in DOX-treated CMs. We found that SIRT3 inhibition abrogates the protective effects of ad-DN-MST1 on cell viability (Online Resource 2—Supplementary Fig. 1e), indicating that MST1 inhibition reduces DOX-induced cytotoxicity partly through SIRT3-dependent mechanisms.

**Fig. 3:**
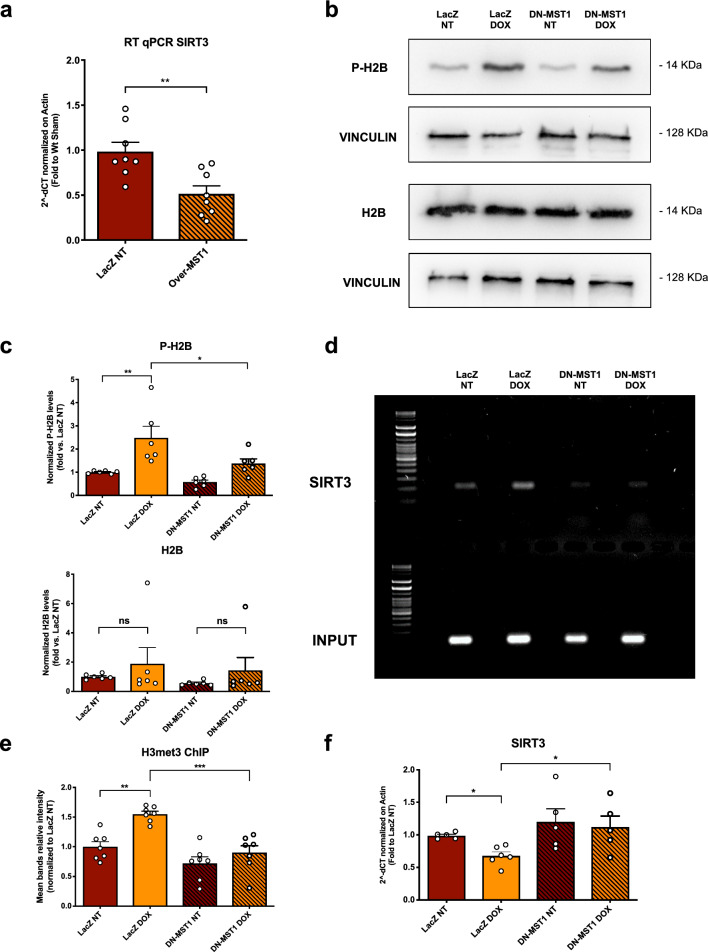
MST1 downregulates SIRT3 through *sirt3* promoter heterochromatinization, **a** SIRT3 mRNA expression profile in cardiomyocytes infected 48 h with an adenovirus overexpressing LacZ or MST1. Data represent mean ± SEM (n = 8 independent samples)**; b**–**c** P_ser14_-H2B and H2B expression profiles after 4 h of treatment with 50 μM of doxorubicin in primary cardiomyocyte cultures infected with ad-LacZ or ad-DN-MST1 for 48 h. Densitometric data normalized by loading control represent mean ± SEM (*n* = 6 independent samples); **d**–**e** sirt3 promoter DNA amplification after chromatin immunoprecipitation (ChIP) targeting trimethyl (Lys27) Histone 3 (H3K27) in H9C2 cardiac cells infected 48 h with an adenovirus overexpressing LacZ or DN-MST1. Data represent mean ± SEM (n = 7 independent replicates from pulled cells); **f** SIRT3 mRNA expression profile in H9C2 cardiac cells infected 48 h with an adenovirus overexpressing LacZ or DN-MST1. Data represent mean ± SEM (n = 5–6 independent samples). *Data were analysed with a two-tailed Student’s t-test (a) or one-way ANOVA with Bonferroni post-hoc test *(**b**–**f**). *P ≤ 0.05; **P ≤ 0.01; ***P ≤ *0.001*

Then, we checked whether MST1 activation suppresses SIRT3 expression levels at a transcriptional level. First, we found that adenoviral-mediated overexpression of wild-type MST1 protein in cardiomyocytes is sufficient to induce SIRT3 mRNA downregulation (Fig. 3a). In addition, DOX treatment increases Ser14 phosphorylation of histone H2B, but this effect is reduced by MST1 inhibition (Fig. 3b, c). Previous work demonstrated that phosphorylation of H2B by MST1 is a histone modification that promotes heterochromatinization by recruiting epigenetic enzymatic modifiers. Therefore, we investigated whether the promoter region of SIRT3 is enriched with epigenetic modifications associated with transcriptional repression in response to DOX. ChIP assay showed that DOX treatment promotes Histone 3 (H3) Lys27 tri-methylation in the promoter region of SIRT3 in H9C2 cardiomyocytes in response to DOX treatment, indicating increased chromatin condensation. Conversely, MST1 inhibition by DN-MST1 overexpression reduces the levels of H3 tri-methylation (Fig. 3d, e). These results were consistent with the observations that SIRT3 mRNA levels are reduced in cells treated with DOX, whereas SIRT3 levels are restored by MST1 inhibition (Fig. 3f). Overall, these data may suggest that MST1 activation by DOX mediates transcriptional inhibition of SIRT3 expression through the induction of epigenetic repressive histone modifications.

### MST1 inhibition abrogates DOX-induced cardiac dysfunction in vivo

**Fig. 4:**
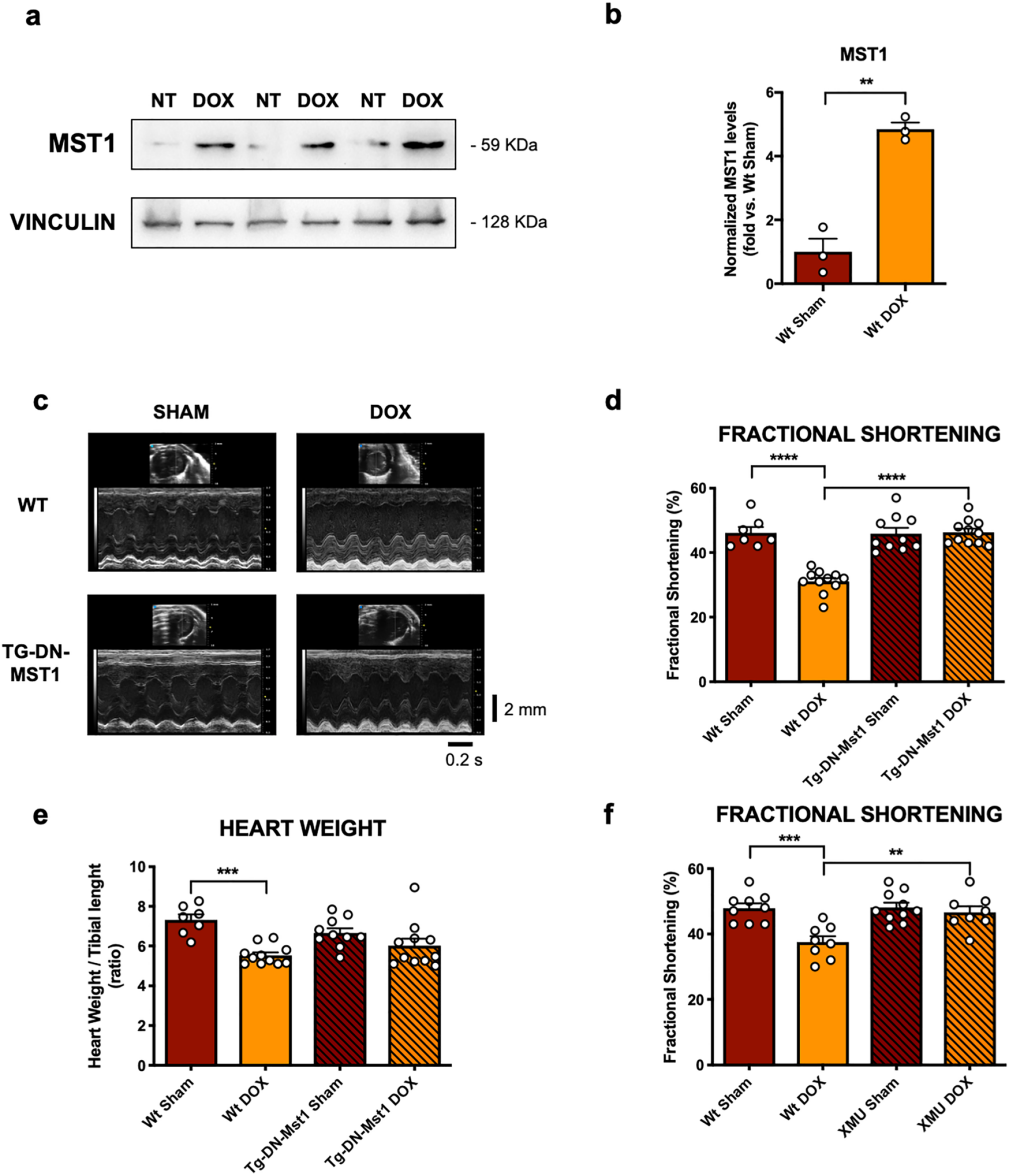
MST1 mediates DOX-induced myocardial dysfunction,. (**a**–**b**) MST1 expression profile after three weeks of treatment with 18 mg/kg of doxorubicin in C57BL/6J mice. Densitometric data normalized by loading control represent mean ± SEM (*n* = 3 independent samples); **c** M-mode echocardiographic analyses after six weeks of treatment with doxorubicin (DOX) in C57BL/6J (wt) and Tg-DN-MST1 mice; **d**–**e** echocardiographic measurements of fractional shortening *(****d****)* and *post-mortem* gravimetric analyses of heart weight/tibial length ratio (**e**) after six weeks of treatment with doxorubicin in C57BL/6J (wt) and Tg-DN-MST1 mice. Data represent mean ± SEM (*n* = 7–11 independent samples); **f** echocardiographic measurements of fractional shortening after six weeks of treatment with doxorubicin in C57BL/6J (wt) with or without the MST1 inhibitor XMU-MP-1 (XMU). Data represent mean ± SEM (*n* = 8–10 independent samples). *Data were analysed with a two-tailed Student’s t-*test (**b**)* or one-way ANOVA with Bonferroni post-hoc test *(**d**–**f**). **P ≤ 0.01; ***P ≤ 0.01; ****P ≤ *0.0001*

We then transferred our in vitro findings to a relevant and validated pre-clinical murine model of DOX-induced cardiotoxicity. We studied the cardiac phenotype of a transgenic C57BL/6J model with cardiomyocyte-specific overexpression of DN-MST1 (Tg-DN-MST1) [14] in response to DOX treatment. Heterozygous Tg-DN-MST1 and wild type (WT) littermate mice were treated with a final dose of 18 mg/kg DOX, which was injected i.p. once a week for three consecutive weeks (three injections of 6 mg/kg, see Material and Methods for details). First of all, we studied MST1 cardiac levels in WT mice after one, three and six weeks of treatment and observed a significant upregulation after three and six weeks from the first injection of DOX (Fig. 4a, b, Online Resource 2—Supplementary Fig. 3a-b).

We confirmed the efficacy of our Hippo-deficient Tg-DN-MST1 mouse model by observing that P-LATS level was increased in response to DOX treatment in WT but not in Tg-DN-MST1 mice heart lysates (Online Resource 2—Supplementary Fig. 3c, d). Of interest, we found that cardiac MST1 accumulates in the cytosol and, more markedly, in the nucleus in response to DOX treatment (Online Resource 2—Supplementary Fig. 4a, b).

We also observed a significant reduction of systolic function in WT mice, as indicated by reduced fractional shortening, after six weeks since the initial injection of DOX. A significant increase in left ventricular end-systolic diameter in WT mice treated with DOX was also observed, whereas left ventricular end-diastolic diameter was unchanged, consistent with a recent onset of systolic dysfunction. Conversely, Tg-DN-MST1 mice do not show an impairment of systolic function or an increased left ventricular end-systolic diameter in response to DOX treatment (Fig. 4c, d, Online Resource 3—Supplementary Table 1), demonstrating that MST1 inhibition abrogates DOX-induced cardiac abnormalities. We also observed a significant reduction of left ventricular wall thickness in WT mice but not in Tg-DN-MST1 mice in response to DOX treatment (Online Resource 3—Supplementary Table 1).

Likewise, gravimetric analyses showed a significant loss of heart mass in WT mice treated with DOX, as compared to untreated mice. In contrast, heart mass is unchanged in Tg-DN-MST1 mice treated with DOX with respect to untreated mice (Fig. 4e).

Similar results were also observed when MST1 was pharmacologically inhibited in mice treated with DOX. Administration of XMU-MP-1 (XMU), a novel pharmacological MST1 enzymatic function inhibitor [15], to C57/BL6J WT mice treated with DOX can abrogate DOX-induced cardiac dysfunction, thereby recapitulating the beneficial effects of DN-MST1 overexpression (Fig. 4f, Online Resource 3—Supplementary Table 2). Overall, these data show that either genetic or pharmacological inhibition of MST1 reduces the development of cardiac dysfunction induced by DOX.

Interestingly, WT mice had increased mortality in response to DOX treatment with respect to untreated animals, but pharmacological MST1 inhibition by XMU-MP-1 administration significantly inhibited it, markedly improving survival. Conversely, Tg-DN-MST1 mice receiving DOX treatment did show increased survival with respect to WT mice, despite a marked attenuation of cardiac dysfunction (Online Resource 2 – Supplementary Fig. 4c).

### MST1 inhibition attenuates DOX-induced cardiac fibrosis and apoptosis

**Fig. 5:**
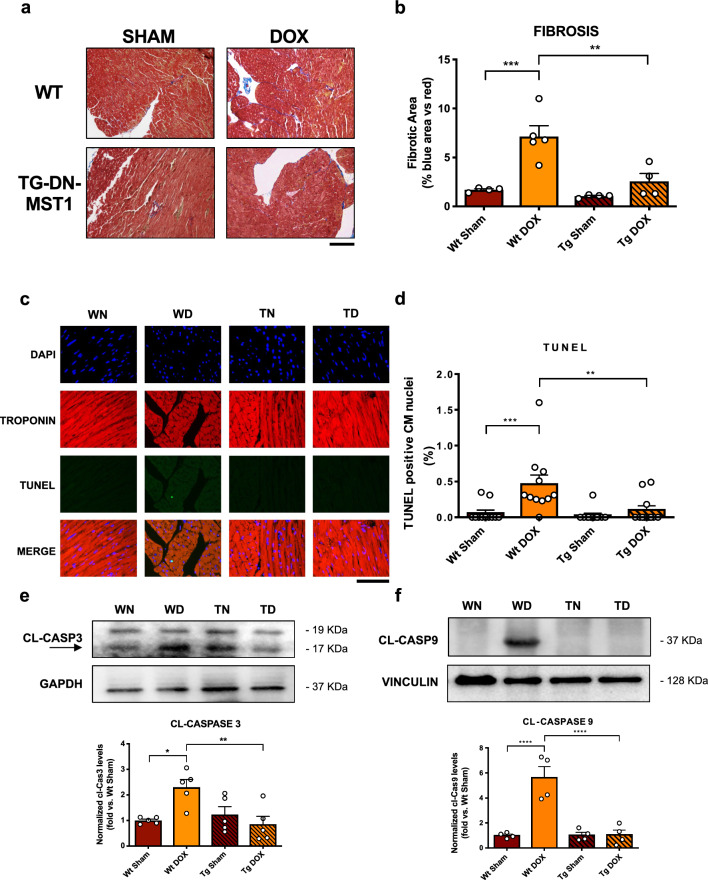
MST1 inhibition protects from DOX-induced cell death and myocardial remodeling in vivo (**a**–**b**) Masson’s trichrome staining of myocardial sections harvested from mice that were treated with 3 injections of doxorubicin with a final cumulative dose of 18 mg/kg, six weeks after the first administration. Data represent mean ± SEM (*n* = 4–5 independent samples) Scale bar = 200 μm; **c**–**d** TUNEL (*green)* staining was also performed in the same conditions, and nuclei were counterstained with DAPI. TUNEL-positive nuclei from Troponin-positive cells (i.e., CM) were counted. Data represent mean ± SEM (*n* = 11 independent samples). Scale bar = 100 μm; **e**–**f** Cl-Caspase 3 and Cl-Caspase 9 expression profiles in myocardial lysates deriving from mice treated with doxorubicin to a final cumulative dose of 18 mg/kg and sacrificed six weeks after the first of three 6 mg/kg injections. The arrow marks the inferior bands as cleaved-Caspase3. Densitometric data normalized by loading control represent mean ± SEM (*n* = 5 (e) and n = 4 (f) independent samples). Data were analysed with one-way ANOVA with Bonferroni post-hoc test. *P ≤ 0.05; **P ≤ 0.01; ***P ≤ 0.001; ****P ≤ *0.0001; ns* = *P* > *0.05. Tg* = *transgenic DN-MST1; NT* = *not-treated (saline-injected) WN* = *wild type not-treated (sham); *WD *wild type doxorubicin-treated, TN* *transgenic not-treated; TD* *transgenic doxorubicin-treated*

We performed ex vivo histological analyses to check whether DOX treatment induces myocardial abnormalities in an MST1-dependent manner. We evaluated cardiac fibrosis through Masson’s Trichrome staining in WT and Tg-DN-MST1 mice with or without DOX treatment and observed that DOX treatment promotes collagen deposition in WT mice. Conversely, cardiac fibrosis is attenuated in heart sections of Tg-DN-MST1 mice treated with DOX with respect to treated WT mice (Fig. 5a, b). We also measured CM cross-sectional area (CSA) and revealed a significant reduction in WT mice in response to DOX treatment, which is abolished in Tg-DN-MST1 mice, as compared to untreated controls (Online Resource 2 – Supplementary Fig. 5).

We also evaluated cardiac apoptosis by performing a TUNEL assay and found a marked increase in TUNEL-positive CM nuclei in DOX-treated WT mice with respect to untreated controls. On the other hand, DN-MST1 overexpression reduces the percentage of TUNEL-positive nuclei in DOX-treated transgenic mice (Fig. 5c, d). These data were also corroborated by the evidence that cardiac cleaved-Caspase 3 and cleaved-Caspase 9 levels are significantly increased in DOX-treated WT mice as compared to untreated animals but not in DOX-treated Tg-DN-MST1 (Fig. 5e, f). Overall, these data indicate that DOX administration provokes myocardial atrophy, cell death and fibrosis through MST1-dependent mechanisms.

### MST1 inhibition preserves cardiac function through SIRT3 activation in vivo

**Fig. 6:**
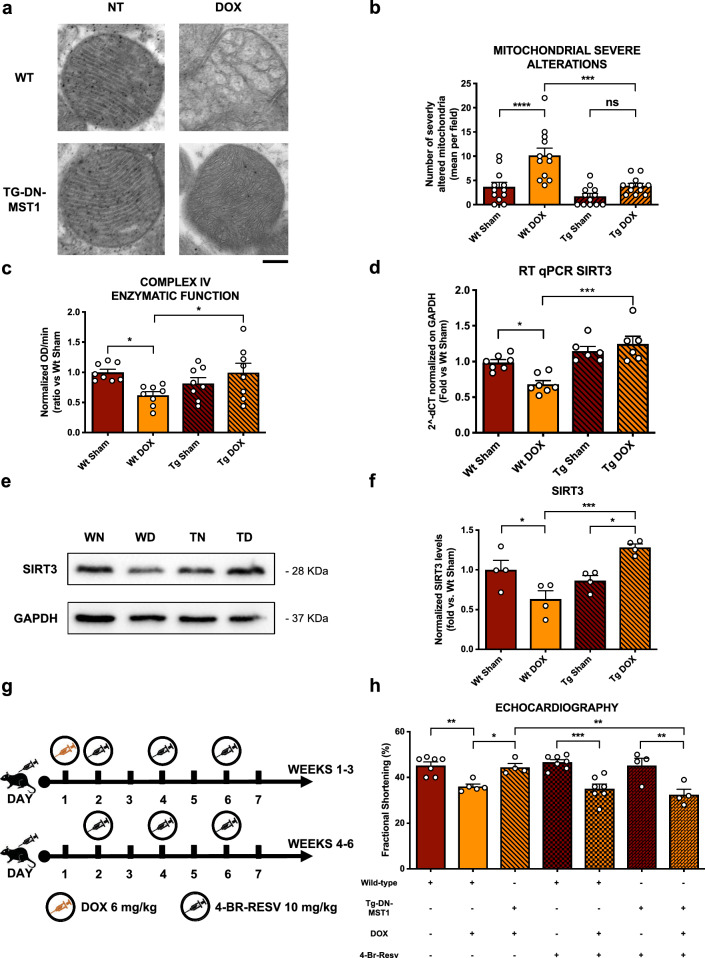
MST1 inhibition reduces DOX-induced cytotoxic mitochondrial alterations by SIRT3 uperegulation in vivo*,* **a** Representative TEM images of myocardial mitochondria from mice treated with DOX, six weeks after the first administration. Scale bar = 0.2 μm; **b** quantification of the number of mitochondria presenting severe alterations per microscopic field*.* Data represent mean ± SEM (*n* = 12 microscopic fields from 3 independent samples); **c** colorimetric evaluation of mitochondrial complex IV enzymatic function measured as –OD/min slope. Data represent mean ± SEM (*n* = 8 independent samples); **d** SIRT3 mRNA expression profile in myocardial lysates deriving from mice treated with doxorubicin reaching a final cumulative dose of 18 mg/kg. Data represent mean ± SEM (*n* = 6–7 independent samples); **e–f** SIRT3 protein expression profile in myocardial lysates deriving from mice that were treated six weeks with doxorubicin reaching a final cumulative dose of 18 mg/kg. Densitometric data normalized by loading control represent mean ± SEM (*n* = 4 independent samples); **g** diagram of the experimental procedure used for the i.p. administration of the SIRT3 inhibitor 4’-Br-Resveratrol together with doxorubicin; **h** functional echocardiographic analyses of the fractional shortening measured on mice treated with both doxorubicin and 4’-Br-Resveratrol. Data represent mean ± SEM (*n* = 4–7 independent samples). Data were analysed with one-way ANOVA with Bonferroni post-hoc test. *P ≤ 0.05; **P ≤ 0.01; ***P ≤ 0.001; ****P ≤ *0.0001; ns* = *not significant (P* > *0.05). Tg* = *transgenic DN-MST1; NT* = *not-treated (saline-injected) WN* = *wild type not-treated (sham); WD* = *wild type doxorubicin-treated; TN* = *transgenic not-treated; TD* = *transgenic doxorubicin-treated; WNB* = *wild-type 4’-Br-resveratrol-treated; WDB* = *wild type doxorubicin- and 4’-Br-resveratrol-treated; TNB* = *transgenic 4’-Br-resveratrol-treated; TDB* = *transgenic doxorubicin- and 4’-Br-resveratrol-treated*

To evaluate the impact of MST1 inhibition on mitochondrial structure and function in response to DOX treatment, we performed transmission electronic microscopy (TEM) analysis in heart samples of WT and Tg-DN-MST1 at baseline and in response to DOX treatment. We found that DOX treatment significantly induces mitochondrial damage in the hearts of WT mice, as indicated by a higher number of mitochondria with deranged cristae morphology and increased mitochondrial cross-sectional area, which denotes swelling. Conversely, the mitochondrial structure is preserved in Tg-DN-MST1 treated with DOX (Fig. 6a, b; Online Resource 2 – Supplementary Fig. 6a–c). Moreover, we measured an increase in mitochondrial mass by COX IV quantification in WT mice that received DOX (Online Resource 2 – Supplementary Fig. 6d-e). This result is in line with the observed autophagy abnormalities in WT mice after three weeks of treatment with DOX. In fact, we found increased levels of both LC3B-II and p62 autophagy markers in WT mice, which together suggest autophagy flux inhibition, in line with previously published evidence [16]. In contrast, no autophagy abnormalities were observed in Tg-DN-MST1 treated with DOX, in accordance with the known pro-autophagic effect of MST1 inhibition [17] (Online Resource 2 – Supplementary Fig. 7a-b). We also found that mitochondrial function is impaired in DOX-treated WT mice with respect to untreated controls, as indicated by reduced mitochondrial complex IV activity, whereas it is preserved in DOX-treated Tg-DN-MST1 mice (Fig. 6c). Overall, these data suggest that MST1 inhibition preserves mitochondrial function and structure in response to DOX treatment.

Then, we wanted to investigate whether SIRT3 is involved in the beneficial effects of MST1 inhibition in vivo*.* We observed a significant reduction of SIRT3 mRNA levels in WT mice treated with DOX, as compared to untreated animals. On the other hand, SIRT3 levels are preserved in Tg-DN-MST1 mice in response to DOX treatment (Fig. 6d). SIRT3 protein levels are also reduced in WT mice treated with DOX, whereas they are preserved in transgenic mice, being even higher than in untreated transgenic controls (Fig. 6e, f). We also measured SIRT3 levels in mitochondrial heart fractions and confirmed that SIRT3 protein levels decrease in WT mice receiving DOX treatment (Online Resource 2 – Supplementary Fig. 8a-b).

Together, these results indicate that MST1 activation in response to DOX treatment contributes to the downregulation of SIRT3 levels in a transcriptional manner, also in vivo.

We then investigated whether pharmacological inhibition of SIRT3 by 4’-Br-Resv administration would neutralize the cardioprotective effects of MST1 inhibition observed in Tg-DN-MST1 mice in response to DOX treatment (Fig. 6g). First, we found that SIRT3 inhibition does not affect basal heart function in both untreated WT and Tg-DN-MST1 mice. In contrast, SIRT3 inhibition abrogates the protective effects of MST1 inhibition in response to DOX treatment since Tg-DN-MST1 mice show a significant decline in systolic function after DOX treatment, which is comparable to the reduction of systolic function observed in DOX-treated WT mice (Fig. 6h; Online Resource 2 – Supplementary Fig. 8c; Online Resource 3 – Supplementary Table 3). These data demonstrate that MST1 inhibition protects cardiac function in response to DOX treatment by preserving SIRT3 levels.

### MST1 is upregulated in the hearts of patients treated with doxorubicin

**Fig. 7:**
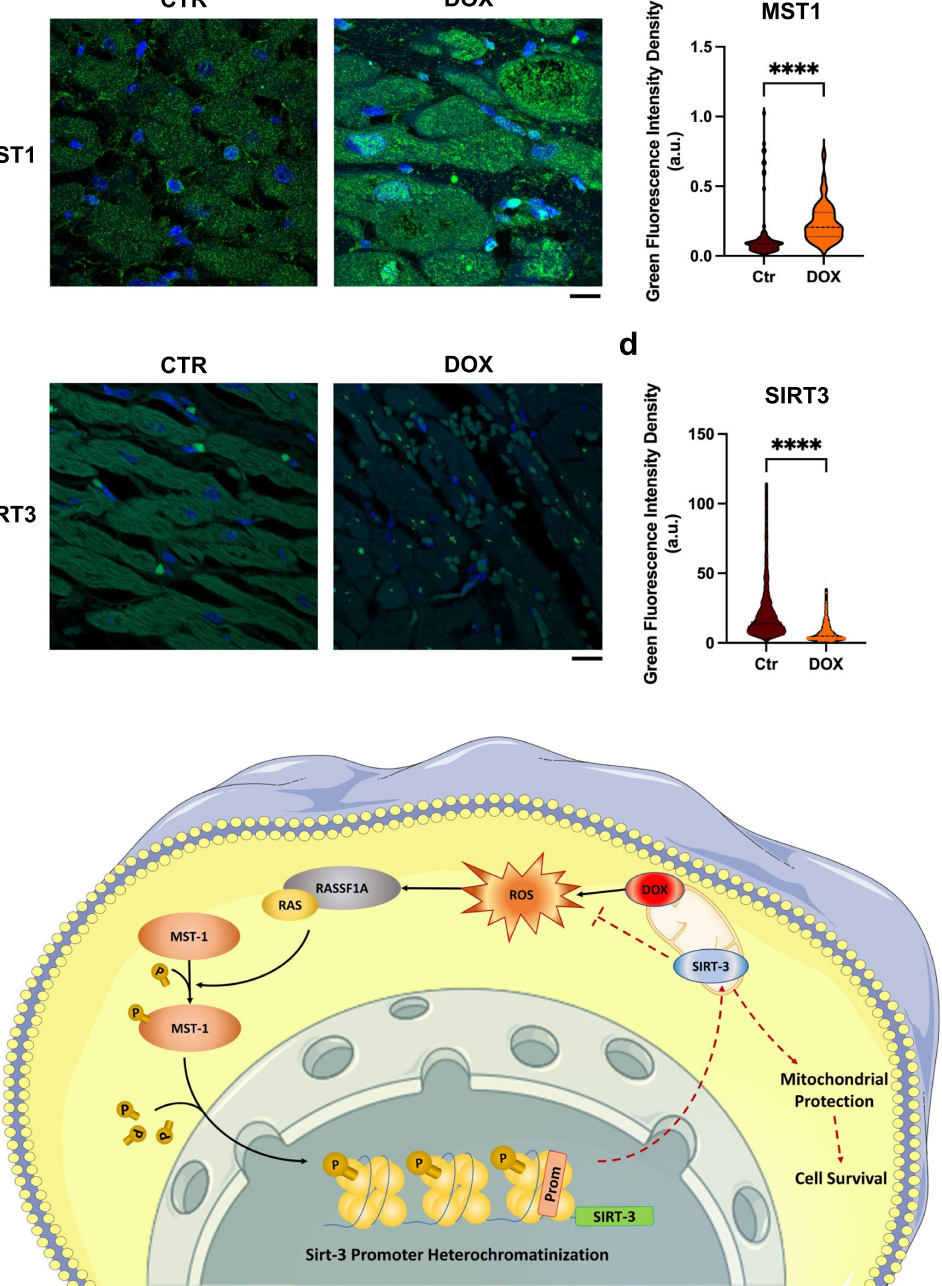
MST1 is upregulated in the heart of DOX-treated patients, (**a**–**d**) Confocal IF analysis of human heart sections from healthy control and patients treated with doxorubicin, stained with an antibody targeting MST1 (**a**) and SIRT3 (**c**). Nuclei were counterstained with DAPI. Scale bar = 10 μm; (**c**, **d**) quantifications of MST1 and SIRT3 mean fluorescence intensity in cardiomyocytes (CMs) from heart sections of control *vs*. DOX-treated subjects. Data represent mean ± SEM (n = 84–123 CMs from three independent patients per group). Statistical significance was assessed with a Mann–Whitney test; **e** graphical abstract of the findings of this study, showing that doxorubicin enhances mitochondrial ROS production, thereby triggering MST1 activation (black arrows). MST1, in turn, stimulates sirt3 promoter heterochromatinization and, therefore, downregulates its expression, impairing SIRT3 pleiotropic mitochondrial protective effects (dashed red lines) and promoting cell death. This image was made in part using tools provided by Servier Medical Arts. Data were analysed with a two-tailed Student’s t-test. ****P ≤ *0.0001* (color figure online)

To translate our findings to a clinical level, we checked the expression levels of MST1 in heart samples derived from patients with cancer and treated with DOX who developed cardiomyopathy (Online Resource 3 – Supplementary Table 4–5). We observed that MST1 is significantly upregulated in the hearts of patients treated with DOX with respect to control patients (who died because of non-cardiac causes) and is mainly localized to nuclei, as observed in histological analysis (Fig. 7a, b, Online Resource 2 – Supplementary Fig. 9). We also found that SIRT3 levels are reduced in patients with DOX-induced cardiomyopathy, as compared to control patients (Fig. 7c, d). This result suggests that MST1 activation and SIRT3 downregulation are involved in the detrimental cardiac effects of DOX treatment in human subjects.

### Pharmacological MST1 inhibition does not interfere with the chemotherapeutic effects of DOX treatment against cancer cells in vitro

Lastly, we conducted additional experiments to evaluate whether inhibition of MST1 would impair the effectiveness of DOX chemotherapeutic effects. We plated MCF-7 breast cancer cells and then treated them with DOX for four hours, with or without XMU-MP-1, a novel MST1 pharmacological inhibitor [15]. Then, we harvested the cells and seeded them at 100 cells/ml to evaluate the clonogenic potential after two weeks. We observed that XMU-MP-1 does not affect the ability of DOX treatment to suppress the clonogenic potential of cancer cells, but it is actually able to reduce clone formation itself even in the absence of DOX treatment (Online Resource 2 – Supplementary Fig. 10a, b).

## Discussion

In recent years, much effort has been devoted to developing new preventive and therapeutic strategies for the management of the cardiotoxic effects of anthracycline-based anticancer therapies [18].

The present work extends the current knowledge about the molecular mechanisms involved in DOX-associated cardiotoxicity, showing for the first time the prominent role of MST1 signaling. We found that DOX treatment significantly activates MST1 signaling in cardiomyocytes both in vitro and in vivo. MST1 inhibition by overexpression of a ‘kinase-dead’ form of this protein reduces cardiomyocyte death and apoptosis and preserves cardiac function in vivo. A similar result was obtained by administrating XMU-MP-1, a pharmacological inhibitor of MST1, supporting the translational potential of our findings. These results are in line with previous work demonstrating the involvement of MST1 in the induction of myocardial injury in response to other stresses such as β1-adrenergic cardiomyopathy [19], chronic myocardial infarction [20] and ischemia/reperfusion injury [21]. Our findings extend this concept by highlighting a novel pathophysiological context in which MST1 plays a major role in cardiomyocyte response to stress.

We observed that the protective effects of MST1 inhibition are associated with a reduction of mitochondrial damage and oxidative stress, indicating that MST1 activation in response to DOX treatment contributes to mitochondrial derangements. This notion is consistent with previous results demonstrating that MST1 translocates to mitochondria in response to oxidative stress and induces mitochondrial-mediated apoptotic cell death by phosphorylating BCL-XL [11].

Our study demonstrates that the beneficial effects of MST1 inhibition in response to DOX treatment are mediated by SIRT3 upregulation. In fact, the beneficial effects of DN-MST1 overexpression on cardiac function are abrogated by concomitant SIRT3 inhibition. SIRT3 is a gene encoding for a mitochondrial deacetylase, whose activity is essential for mitochondrial integrity, function and ROS handling. It was previously demonstrated that SIRT3 is downregulated in response to DOX treatment, thereby promoting the development of cardiomyopathy [12]. We believe that our paper significantly extends this evidence by demonstrating that MST1 activation is responsible for SIRT3 downregulation, apparently through transcriptional mechanisms. In fact, DOX treatment reduces SIRT3 mRNA levels in the heart, which are reversed by DN-MST1 overexpression. In this line of evidence, MST1 inhibition reverses the effects of DOX treatment on SIRT3 mRNA levels, whereas MST1 overexpression is sufficient to induce a decrease of SIRT3 mRNA levels in cardiomyocytes in vitro. Interestingly, we found that DOX induces H2B Ser14 phosphorylation, a known repressive histone modification, and enriches H3 Lys27 tri-methylation at the level of the SIRT3 promoter in an MST1-dependent manner. These results may suggest that MST1 represses SIRT3 gene expression through the induction of epigenetic modifications, which may reduce SIRT3 promoter accessibility to the transcriptional machinery. Future studies are warranted to corroborate this hypothesis.

Previous evidence also showed that SIRT3 stimulates autophagy, whereas MST1 inhibits it. Autophagy is impaired in cardiomyocytes by DOX treatment [16]. Our data suggest that autophagy flux is inhibited in our model and that DN-MST1 preserves it in response to DOX, fully confirming previous evidence [17]. This result further increases the complexity of our understanding of CM responses to cytotoxic stresses, such as DOX, and how deeply the different pathways associated with DCM that were identified over the years are interconnected.

Our findings are also relevant to human disease since we found a marked MST1 activation and SIRT3 reduction in myocardial specimens of patients with cancer treated with DOX-based therapy a few years earlier. This result suggests that MST1 activation in cardiomyocytes persists for a long time after chemotherapy, likely because of the onset of a vicious cycle characterized by mitochondrial damage and marked ROS production activating MST1, which in turn further deteriorates mitochondrial damage and oxidative stress. More remarkably, our results in human heart samples suggest that new therapies targeting MST1 may be potentially helpful for improving the cardiac prognosis of cancer patients treated with DOX. Our data on the improved survival of mice receiving DOX together with the MST1/2-inhibitor ‘XMU-MP-1’ are encouraging in this sense. Of note, this positive effect on survival was not observed in our model of cardiac-specific Tg-DN-MST1 mice. We hypothesize that this difference may be due to the multi-organ protective effect that is granted by XMU-MP-1 administration. Future investigations are warranted to test this hypothesis.

We also conducted in vitro experiments to test whether MST1 inhibition may interfere with the anticancer effects of DOX. In fact, Hippo signaling is known to exert oncosuppressive activities. However, our experiments on MCF-7 human breast cancer cells treated with DOX and XMU-MP-1, a novel MST1 pharmacological inhibitor, showed that MST1 inhibition does not only affect DOX cytotoxicity at all, but it reduces the clonogenic potential of cancer cells, also in the absence of DOX treatment. This observation aligns with studies showing that mice with MST1 or MST2 gene deletion do not show spontaneous cancer formation [22, 23].

This study has some limitations to be mentioned. We apparently used high doses of DOX for our in vitro studies. We chose this DOX concentration based on the evidence that the drug progressively accumulates inside the cells, reaching intracellular concentrations significantly higher than circulating ones. Therefore, we thought that it was more clinically relevant to test doses in the micromole range. In addition, the human study was conducted on a relatively small number of patients. However, a comprehensive analysis was performed in heart sections, which was sufficient to see significant differences. Finally, the exact molecular mechanism through which MST1 inhibits SIRT3 expression was not fully elucidated in this study and warrants clarification in future, more focused studies.

In conclusion, we propose a mechanistic model underlying DOX-induced cardiac injury in which MST1 is activated and epigenetically downregulates SIRT3 transcription, thereby leading to mitochondrial damage and cardiac dysfunction (Fig. 7e). Moreover, we show for the first time that inhibiting MST1 with pharmacological agents, such as XMU-MP-1, represents a novel translational approach to prevent the occurrence of DOX-induced cardiomyopathy.

## Supplementary Information

Below is the link to the electronic supplementary material.

Supplementary file1 (DOCX 20 KB)

Supplementary file2 (DOCX 11426 KB)

Supplementary file3 (DOCX 798 KB)

## Data Availability

The data underlying this article will be shared on reasonable request to the corresponding authors.

## Abbreviations

Ad: Adenovirus
DCM: Doxorubicin-induced cardiomyopathy
DN-MST1: Overexpressing the K59R dominant-negative form of MST1
DOX: Doxorubicin
MST1: Mammalian ste20 kinase
OXPHOS CI: Oxidative phosphorylation complex I
ROS: Reactive oxygen species
SIRT3: Sirtuin 3
TEM: Transmission electron microscopy

## References

[1] Jordan MA (2002). Mechanism of action of antitumor drugs that interact with microtubules and tubulin. Curr Med Chem Anticancer Agents.

[2] Takemura G, Fujiwara H (2007). Doxorubicin-induced cardiomyopathy from the cardiotoxic mechanisms to management. Prog Cardiovasc Dis.

[3] McGowan JV (2017). Anthracycline chemotherapy and cardiotoxicity. Cardiovasc Drugs Ther.

[4] Yu FX, Zhao B, Guan KL (2015). Hippo pathway in organ size control, tissue homeostasis, and cancer. Cell.

[5] Del Re DP (2013). Yes-associated protein isoform 1 (Yap1) promotes cardiomyocyte survival and growth to protect against myocardial ischemic injury. J Biol Chem.

[6] Xin M (2011). Regulation of insulin-like growth factor signaling by Yap governs cardiomyocyte proliferation and embryonic heart size. Sci Signal.

[7] Ikeda S (2019). Hippo deficiency leads to cardiac dysfunction accompanied by cardiomyocyte dedifferentiation during pressure overload. Circ Res.

[8] Fioriniello S (2020). MeCP2 and major satellite forward RNA cooperate for pericentric heterochromatin organization. Stem Cell Reports.

[9] Zaglia T (2016). Optimized protocol for immunostaining of experimental GFP-expressing and human hearts. Histochem Cell Biol.

[10] Singal PK, Iliskovic N (1998). Doxorubicin-induced cardiomyopathy. N Engl J Med.

[11] Del Re DP (2014). Mst1 promotes cardiac myocyte apoptosis through phosphorylation and inhibition of Bcl-xL. Mol Cell.

[12] Cheung KG (2015). Sirtuin-3 (SIRT3) protein attenuates doxorubicin-induced oxidative stress and improves mitochondrial respiration in H9c2 cardiomyocytes. J Biol Chem.

[13] Yao S, Yan W (2018). Overexpression of Mst1 reduces gastric cancer cell viability by repressing the AMPK-Sirt3 pathway and activating mitochondrial fission. Onco Targets Ther.

[14] Yamamoto S (2003). Activation of Mst1 causes dilated cardiomyopathy by stimulating apoptosis without compensatory ventricular myocyte hypertrophy. J Clin Invest.

[15] Triastuti E (2019). Pharmacological inhibition of Hippo pathway, with the novel kinase inhibitor XMU-MP-1, protects the heart against adverse effects during pressure overload. Br J Pharmacol.

[16] Li M (2018). Phosphoinositide 3-kinase gamma inhibition protects from Anthracycline cardiotoxicity and reduces tumor growth. Circulation.

[17] Maejima Y (2013). Mst1 inhibits autophagy by promoting the interaction between Beclin1 and Bcl-2. Nat Med.

[18] Wenningmann N (2019). Insights into doxorubicin-induced cardiotoxicity: molecular mechanisms, preventive strategies, and early monitoring. Mol Pharmacol.

[19] Lee GJ (2015). Mst1 inhibition rescues beta1-adrenergic cardiomyopathy by reducing myocyte necrosis and non-myocyte apoptosis rather than myocyte apoptosis. Basic Res Cardiol.

[20] Odashima M (2007). Inhibition of endogenous Mst1 prevents apoptosis and cardiac dysfunction without affecting cardiac hypertrophy after myocardial infarction. Circ Res.

[21] Nakamura M (2016). Mst1-mediated phosphorylation of Bcl-xL is required for myocardial reperfusion injury. JCI Insight.

[22] Song H (2010). Mammalian Mst1 and Mst2 kinases play essential roles in organ size control and tumor suppression. Proc Natl Acad Sci U S A.

[23] Zhou D (2009). Mst1 and Mst2 maintain hepatocyte quiescence and suppress hepatocellular carcinoma development through inactivation of the Yap1 oncogene. Cancer Cell.

