# Supplementary material for: MST1 mediates doxorubicin-induced cardiomyopathy by SIRT3 downregulation
Source: Cell Mol Life Sci. 2023 Aug 11;80(9):245. doi: 10.1007/s00018-023-04877-7 (PMC10421787; doi:10.1007/s00018-023-04877-7)
Supplement: Supplementary file 1 — Supplementary file1 (DOCX 20 KB) [file 18_2023_4877_MOESM1_ESM.docx]

**Supplementary Methods**

**Adenovirus**

Adenoviruses (AD-LACZ, AD-MST1, AD-DN-MST1) were constructed as previously described ^11^ and amplified in HEK-293 cells.

**Cardiomyocyte isolation and culture**

Hearts were harvested from neonatal rats 1-3 days after delivery, atria were removed, and the ventricles were minced in small pieces in PBS at room temperature. Then, we used a commercial kit to digest the tissue, obtain a single cell suspension (Miltenyi Biotec 130-098-373), and negatively select cardiomyocytes through an antibody-based chromatographic column (Miltenyi Biotec 130-105-420) following the manufacturer instructions. Plates were coated with 1% gelatin (Sigma G1890), and cardiomyocytes were diluted 10^6^/ml in custom-made ‘seeding medium’ (10% horse serum, 100µM BrdU). 24 hrs later, cells have stably adhered, and the medium is replaced with a serum-free ‘cardiomyocyte medium’ (CM medium). This medium is reconstituted from lyophilized DMEM/F-12 (Sigma D0547) and enriched with 0.72 g/l glucose (Sigma # G5400), 0.33 g/l sodium pyruvate (Fisher # BP356), 0.017 g/l ascorbic acid (Gibco # 13080-23), 2 µl selenite 0.2 M (Sigma # S5261), 0.004 g/l transferrin (Sigma # T3309), 2 g/l BSA fraction V (Amresco # 0332), 3.57 g/l HEPES (Amresco # 0511), 2.43 g/l sodium bicarbonate (Sigma # S6014) and 10 ml penicillin-streptomycin (Gibco 15070063).

**Clonogenesis**MCF-7 breast cancer immortalized cells were treated at 80-90% confluence with 0.1 µM of DOX with or without Mst1 inhibitor XMU-MP-1 (Sigma) at 1.5 µM for 24 hours. Then, cells were detached using trypsin and seeded on uncoated Petri 60 mm dishes at a concentration of 100 cells/ml. After 14 days, colonies were fixed with PFA 4% and stained with Giemsa.

**Complex IV activity**

Primary rat cardiomyocytes were infected with ad-LACZ or ad-DN-MST1 for 48 hrs and then treated with 50 µM of DOX for 12 hrs. Then, cells were scraped in PBS with protease inhibitors and centrifuged at 300 g for 5 mins. Cells were resuspended and lightly lysed with a commercially available kit to extract mitochondria. Following the manufacturer’s instructions, the mitochondrial lysate was put in separate wells of a commercially available strip coated with an anti-Complex IV antibody in triplicate. Then, a colorimetric substrate including Cytochrome C was added, and absorbance was kinetically recorded every minute at 37°C for 2 hours at 450 nm. The initial slope of the curve [i.e. (OD_15min_-OD_5min_)/10 min] was then calculated and compared to evaluate the enzyme’s functionality.

**Electron Microscopy**

Myocardium samples were fixed in glutaraldehyde 2.5% in PBS 0.1 M at pH 7.4 for at least 48 h at 4 °C and then rinsed with PBS. Similarly, cardiomyocytes were gently harvested with Accutase™, pelleted and fixed as detailed above. Osmium tetroxide 1.33% (Agar Scientific, Stansted, UK) was used for 2 hours as the post-fixation solution. Samples were washed twice for 20 mins to remove osmium tetroxide solution residuals. Dehydration steps in increasing ethanol dilution (30%, 70%, 95%, 3x(100%) v/v) were then performed. The substitution procedure of ethanol with propylene oxide was carried on (BDH Italia, Milan, Italy). A mixture of 50:50 propylene oxide and epoxy resin Agar 100 (SIC, Rome, Italy) was prepared, and the samples were left overnight at 25°C. Then, samples were embedded in epoxy resin Agar 100 and put on a stove at 60C° for 48h. Epoxy resin blocks were cut into semithin sections (1 µm thick). They were collected on glass slides and stained with Azur II to perform light microscope explorative observations (Carl Zeiss Axioskop‐40, Zeiss, Germany). Later, ultrathin sections (80–90 nm) for TEM observations were cut using an ultramicrotome (Leica EM UC6, Vienna, Austria). Ultrathin sections were collected on 200‐mesh copper grids (Assing, Rome, Italy) stained with Uranyless© solution and lead citrate. Imaging was performed using a transmission electron microscope set with an accelerating voltage of 60 kV (Carl Zeiss EM10, Thornwood, NY), as previously described [^9^](#_ENREF_9)^,^ [^10^](#_ENREF_10). Images were acquired with a CCD digital camera (AMT CCD, Deben UK Ltd, Suffolk, UK).

Morphological analysis was conducted using 13 microscopic fields (original magnification 10900X) for each sample. Measurements were made with the Image J image analysis software. The number of mitochondria was counted, and single-mitochondrion cross-sectional area was measured in each microscopic field. The morphology of the mitochondria was evaluated using a score of 0= no alteration, 1 mild alteration (clearing matrices, small stack of abnormally osmiophilic closely apposed cristae), 2 severe alteration/degeneration (large stack of abnormally osmiophilic closely apposed cristae, loss of cristae, large vacuoles, notorious myelin figures).

**Gravimetric Studies**

The whole heart, left ventricle, right ventricle and lungs were washed in PBS to remove the blood and were then dried and weighed. The tibial length was then measured and used to normalize the gravimetric measurement to the effective size of the mouse.

***In vivo* treatment, echocardiography and tissue harvesting**

Littermate mice were injected with DOX (Sigma) i.p. 2 mg/ml at days 0, 7 and 14 to a final cumulative dose of 18 mg/kg. After six weeks from the first injection, mice were subjected to blinded echocardiographic structural (measurement of the anterior wall, posterior wall and septum) and functional (fractional shortening) analyses with a VEVO 3100 (Visualsonics®) using an mx550d probe. Mice were anaesthetized with 2.5% avertin (12ul/g body weight, i.p.). We use avertin since it usually does not induce significant cardiodepressant effects, as compared to ketamine combinations, such as ketamine+xylazine. Before the end of the anaesthesia, mice were euthanised and tissue was harvested for post-mortem analyses.

**Masson’s trichrome staining**

A ring-shaped section of the freshly harvested heart was immediately fixed in 4%PFA, included in paraffin and then sliced into histological sections. Samples were gradually rehydrated and sequentially stained with Weigert's iron hematoxylin for 10 minutes, then in Biebrich scarlet-acid fuchsin solution for 15 minutes and finally in the phosphomolybdic-phosphotungstic acid solution for 15 minutes. Each of these steps was followed by a 15 minute-step of rinsing in warm water and 10 minutes of washing in flowing tap water. Then, the samples were dehydrated in 95% ethanol, cleared in xylene and mounted with a resinous medium. Images were acquired with a D-sight microscope (Menarini) in bright field. Fibrosis was evaluated using ImageJ to perform a color deconvolution and measure the ratio between blue and red-colored areas. Cross-sectional area (CSA) was measured by manually delining each fiber section and calculating the defined area using ImageJ.

**MitoSOX™ and MitoTracker™ staining**

Cardiomyocytes were plated on 8-well chamberslide, grown for 4-6 days and then infected for 48hrs with ad-DN-MST1 or ad-LACZ. Cells were treated for 4 hrs with 50 µM of DOX in CM medium, then replaced with 5 µM of MitoSOX™ dye in CM medium. After 10 minutes of incubation at 37°C, the dye was removed, and nuclei were counterstained with Hoechst (Thermo 33342). Images were acquired with a Nikon Eclipse Ti microscope at the TRITC spectrum of fluorescence (532 nm).

**MTS assay**

Cardiomyocytes were plated on a 96-multiwell plate, grown for 4-6 days and then infected for 48 hrs with ad-DN-MST1 or ad-LACZ. Cells were treated for 24 hrs with 50 µM of DOX in CM medium. After this time, the medium was replaced with 100 µl/well CM medium plus 20 µl of MTS reagent (Promega G3582), following the manufacturer’s instructions. Cells were incubated at 37°C for 2 hrs, and then the absorbance was read at 492 nm with a Sunrise Basic TECAN ELISA plate reader.

**RT-qPCR**

Total RNA was isolated with a commercial kit (RNeasy kit, Qiagen) and quantified using NanoDrop One (Thermo Scientific). Then, cDNA was obtained and amplified using a commercial kit (SensiMix SYBR Hi-ROX kit, Bioline). It was amplified by using a SYBR Green PCR Master Mix 4309155 (Applied Biosystems) with the 7900HT Fast Real-Time PCR System (Applied Biosystems) for 40 cycles according to the following protocol: 95 °C for 15 seconds, 60 °C for 10 seconds, 72 °C for 30 seconds. The reaction products were analyzed by SDS 2.1.1 Software (Applied Biosystems, Cheshire, UK).

SIRT3 FW: 5’-ccaatgtcgctcactacttc-3’

SIRT3 RV: 5’-ggataccagatgctctctca-3’

GAPDH FW: 5’-aacgaccccttcattgacctc-3’

GAPDH RV: 5’-ccttgactgtgccgttgaact-3’

**TUNEL staining**

*In vitro*, cardiomyocytes were plated on 8-well chamberslide, grown for 4-6 days and then infected for 48 hrs with ad-DN-MST1 or ad-LACZ. Then, cells were treated with 50 µM of DOX in CM medium for 24 hrs. Then, cells were fixed with freshly prepared 4% PFA, and TUNEL assay was performed with a commercial kit following the manufacturer's instructions (Sigma 11684795910) and nuclei were counterstained with Hoechst (Thermo 33342). Troponin T was used to highlight cardiomyocytes (Thermo MA5-12960). Images were acquired with a Nikon Eclipse Ti microscope at the FITC spectrum of fluorescence (488 nm).

*In vivo*, a ring-shaped section of the freshly harvested heart was immediately included in OCT and frozen. Then, histological samples were prepared and fixed with 4% PFA. TUNEL assay was performed with a commercial kit following the manufacturer's instructions (Sigma 11684795910), and nuclei were counterstained with Hoechst (Thermo 33342). Troponin T was used to highlight cardiomyocytes (Thermo MA5-12960). Images were acquired with a Nikon Eclipse Ti microscope at the FITC spectrum of fluorescence (488 nm).

**Western Blot**

The culture medium was removed, and the culture plates were washed with PBS. Cells were scraped in ice-cold RIPA lysis buffer (TRIS (pH 7.4) 50 mM, NaCl 150 mM, EDTA 1mM, TritonX 1%, glycerol 10% in dd-H2O), supplemented with phosphatases (Sigma 04906845001) and proteases (Sigma 11873580001) inhibitors. Similarly, whole hearts were homogenized in 200 µl of RIPA lysis buffer with a mortar. The derived homogenates were inverted for 1 hour at 4°C and then clarified by centrifugation at 14460 g for 25 minutes. Supernatants were collected and mixed with 4x Laemmli buffer (Bio-Rad 1610747) and β-mercaptoethanol (2,5%). Samples were boiled for 8 minutes (except the samples to be tested for OXPHOS, following the manufacturer’s instructions). Protein concentration was determined by Bradford colorimetric assay (Bio-Rad 5000001) and then separated using 6 to 15% SDS-PAGE and electro-transferred to polyvinylidene difluoride membranes. Membranes were blocked in 5% non-fat dry milk powder in 0.05% Tween 20 TBS for 90 minutes, and then incubation was performed overnight with the previously mentioned primary antibodies. HRP-conjugated mouse (CS-7076) and rabbit (CS-7074) secondary antibodies were diluted at 1:2000 in blocking buffer and incubated with membranes for 90 minutes. The membranes were developed by an enhanced chemiluminescence kit (Amersham ECL Select™ RPN2235). Acquisition and densitometry analysis was performed by ChemiDoc and Image Lab 5.2.1 (Bio-Rad).
