# Supplementary material for: MST1 mediates doxorubicin-induced cardiomyopathy by SIRT3 downregulation
Source: Cell Mol Life Sci. 2023 Aug 11;80(9):245. doi: 10.1007/s00018-023-04877-7 (PMC10421787; doi:10.1007/s00018-023-04877-7)
Supplement: Supplementary file 2 — Supplementary file2 (DOCX 11426 KB) [file 18_2023_4877_MOESM2_ESM.docx]

**SUPPLEMENTARY FIGURE LEGENDS**

**Supplementary Figure 1**

(**a-b**) P-LATS and LATS expression from cardiomyocyte cultures infected with ad-LacZ or ad-DN-MST1 for 48 hours, then treated with doxorubicin (50 μM) for 4 hours. Densitometric data normalized by loading control represent mean ± SEM (n = 5 independent samples); **(c)** TUNEL (*green)* staining of cardiomyocyte cultures infected with ad-LacZ or ad-DN-MST1 for 48 hours, and then treated with doxorubicin (50 μM) for four hours. Nuclei were counterstained with DAPI (blue) and cardiomyocytic cytoplasm with Troponin T (red). Representative images (*n* = 4 independent samples). Scale bar = 30 μm**; (d)** Quantification of cl-Caspase 3 protein expression profile from cardiomyocyte cultures infected with ad-LacZ or ad-DN-MST1 for 48 hours, then treated with doxorubicin (50 μM) for 4 hours. Densitometric data normalized by loading control represent mean ± SEM (n = 5 independent samples); **(e)** MTS colorimetric assay of cardiomyocyte cultures infected with ad-LacZ or ad-DN-MST1 for 48 hours and then treated with doxorubicin (50 μM) and 0.2 mM 4’-Br-Resveratrol (R) for 4 hours. Data represent mean ± SEM (*n* = 8 independent samples). *Data were analysed with one-way ANOVA with Bonferroni post-hoc test. *P ≤ 0.05; **P ≤ 0.01; ****P ≤ 0.0001; ns = not significant (P > 0.05).*

**Supplementary Figure 2**

**(a)** quantification of mitochondrial area from cardiomyocyte cultures infected with ad-LacZ or ad-DN-MST1 for 48 hours, then treated with doxorubicin (50 μM) for 4 hours. Data represent mean ± SEM (n = 100 mitochondria from 4 independent samples); **(b)** mitochondrial numerosity per microscopic field. Data represent mean ± SEM (n = 10 microscopic fields from 4 independent samples); **(c)** MitoSOX (red) staining of cardiomyocyte cultures infected with ad-LacZ or ad-DN-MST1 for 48 hours, and then treated with doxorubicin (50 μM) for four hours. Mitochondria were also stained with the MitoTracker dye (green). White arrows indicate areas of high dye colocalization (yellow). Representative images (*n* = 4 independent samples). Scale bar=30 μm; **(d-e)** OXPHOS complex III/IV and V expression profile from cardiomyocyte cultures infected with ad-LacZ or ad-DN-MST1 for 48 hours, then treated with doxorubicin (50 μM) for four hours. Densitometric data normalized by loading control represent mean ± SEM (n = 6 independent samples). *Data were analysed with one-way ANOVA with Bonferroni post-hoc test. ****P ≤ 0.0001; ns = not significant (P > 0.05).*

**Supplementary Figure 3**

**(a-b)** MST1 expression profile in cardiac lysates after one, three and six weeks of treatment with 18 mg/kg of doxorubicin in C57BL/6J mice. Densitometric data normalized by loading control represent mean ± SEM (n = 7 independent samples). **(c-d)** P-LATS and LATS expression profiles in cardiac lysates after three weeks of treatment with 18 mg/kg of doxorubicin in C57BL/6J mice. Densitometric data normalized by loading control represent mean ± SEM (n = 3-4 independent samples). *Data were analysed with a two-tailed Student’s t-test (a) or one-way ANOVA with Bonferroni post-hoc test. *P<0.05; **P ≤ 0.01. Tg=transgenic DN-MST1; NT=not-treated (saline-injected) WN=wild type not-treated (sham); WD=wild type doxorubicin-treated; TN=transgenic not-treated; TD=transgenic doxorubicin-treated.*

**Supplementary Figure 4**

**(a-b)** MST1 expression profile in cytosolic and nuclear cardiac fraction after three weeks of treatment with 18 mg/kg of doxorubicin in C57BL/6J mice. Densitometric data normalized by loading control represent mean ± SEM (n = 4-5 independent samples)**; (c)** Kaplan-Meier survival analysis (censoring point: 42 days) of mice receiving 18 mg/kg of doxorubicin (WN: c57BL/6J sham-treated mice; WD: c57BL/6J DOX-treated mice; TN: Tg-DN-MST1 sham-treated mice; TD: Tg-DN-MST1 DOX-treated mice; XN: c57BL/6J sham-treated mice that received XMU-MP-1; XD: c57BL/6J DOX-treated mice that received XMI-MP-1); NWN: nuclear fraction of WN; NWD: nuclear fraction of WD; CWN: cytosolic fraction of WN; CWD: cytosolic fraction of WD.  *Data were analysed with a two-tailed Student’s t-test (a-b) or a Breslow-Wilcoxon test (c). *P<0.05; **P ≤ 0.01.*

**Supplementary Figure 5**

Quantification of the average cardiac myofiber cross-sectional area from histologic myocardial sections harvested from mice that were treated with 3 injections of doxorubicin with a final cumulative dose of 18 mg/kg, 6 weeks after the first administration that were processed with Masson’s trichrome staining. Data represent mean ± SEM (*n* = 4 independent samples). *Data were analysed with one-way ANOVA with Bonferroni post-hoc test.
*P ≤ 0.05.*

**Supplementary Figure 6**

**(a)** Representative images of myocardial mitochondria from DOX-treated WT and transgenic mice - low magnification (1250x). White arrows point at particularly deranged mitochondria; **(b)** Quantification of mitochondrial numerosity (n = 13 microscopic fields) and **(c)** mean mitochondrial area (*n* = 43 measured mitochondria) from three independent myocardial samples observed with TEM. Data represent mean ± SEM; **(d-e)** COX-IV expression profile in cardiac lysates after six weeks of treatment with 18 mg/kg of doxorubicin in C57BL/6J mice. Densitometric data normalized by loading control represent mean ± SEM (n = 8 independent samples).*Data were analysed with one-way ANOVA with Bonferroni post-hoc test. *P ≤ 0.05; ****P ≤ 0.0001.*

**Supplementary Figure 7**

**(a-b)** LC3B-II and p62 expression profile in cardiac lysates after six weeks of treatment with 18 mg/kg of doxorubicin in C57BL/6J mice. Densitometric data normalized by loading control represent mean ± SEM (n = 5-6 independent samples). *Data were analysed with a two-tailed Student’s t-test. *P ≤ 0.05; **P ≤ 0.01; ***P ≤ 0.001. Tg=transgenic DN-MST1; NT=not-treated (saline-injected) WN=wild type not-treated (sham); WD=wild type doxorubicin-treated; TN=transgenic not-treated; TD=transgenic doxorubicin-treated.*

**Supplementary Figure 8**

**(a-b)** SIRT3 expression profile in mitochondrial cardiac fraction after six weeks of treatment with 18 mg/kg of doxorubicin in C57BL/6J mice. Densitometric data normalized by loading control represent mean ± SEM (n = 7 independent samples)**; (c)** M-mode echocardiographic analyses after treatment with doxorubicin 18 mg/kg and/or 4’-Br-Resveratrol 30 mg/kg/week in C57BL/6J and Tg-DN-MST1 mice. Representative images of 4–7 independent samples. *Data were analysed with a two-tailed Student’s T-test. *P ≤ 0.05*

**Supplementary Figure 9**

Quantification of CMs showing MST1 nuclear localization in heart sections from control (CTR) vs. DOX-treated subjects. Data represent mean ± SEM (n=6 microscopic fields from three independent patients per group). *Data were analysed with a two-tailed Student’s T-test. **P ≤ 0.01*

**Supplementary Figure 10**

(**a-b**) Clonogenesis assay of MCF7 human breast carcinoma cells treated with 0.1 or 1 μM doxorubicin and/or 1.5 XMU-MP-1, a pharmacological MST1-inhibitor, for 24 hours. Data represent mean ± SEM (*n* = 7 independent samples).*Groups with and without XMU-MP-1 were analysed separately for statistical significance with a two-tailed Student’s T-test. *P ≤ 0.05; ***P ≤ 0.001;
****P ≤ 0.0001.*

**SUPPLEMENTARY FIGURES**

**
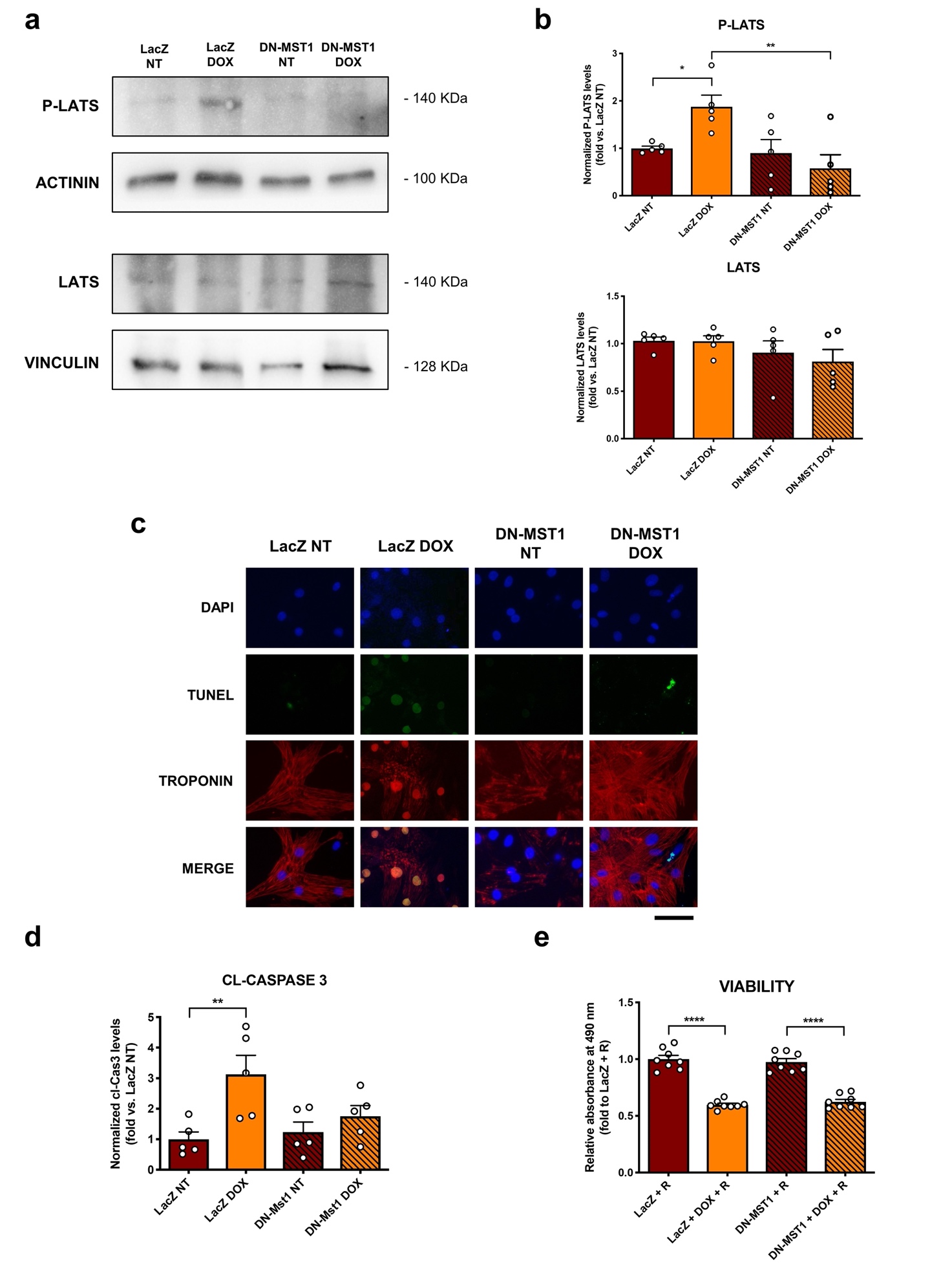
**

**Supplementary Figure 1**

**
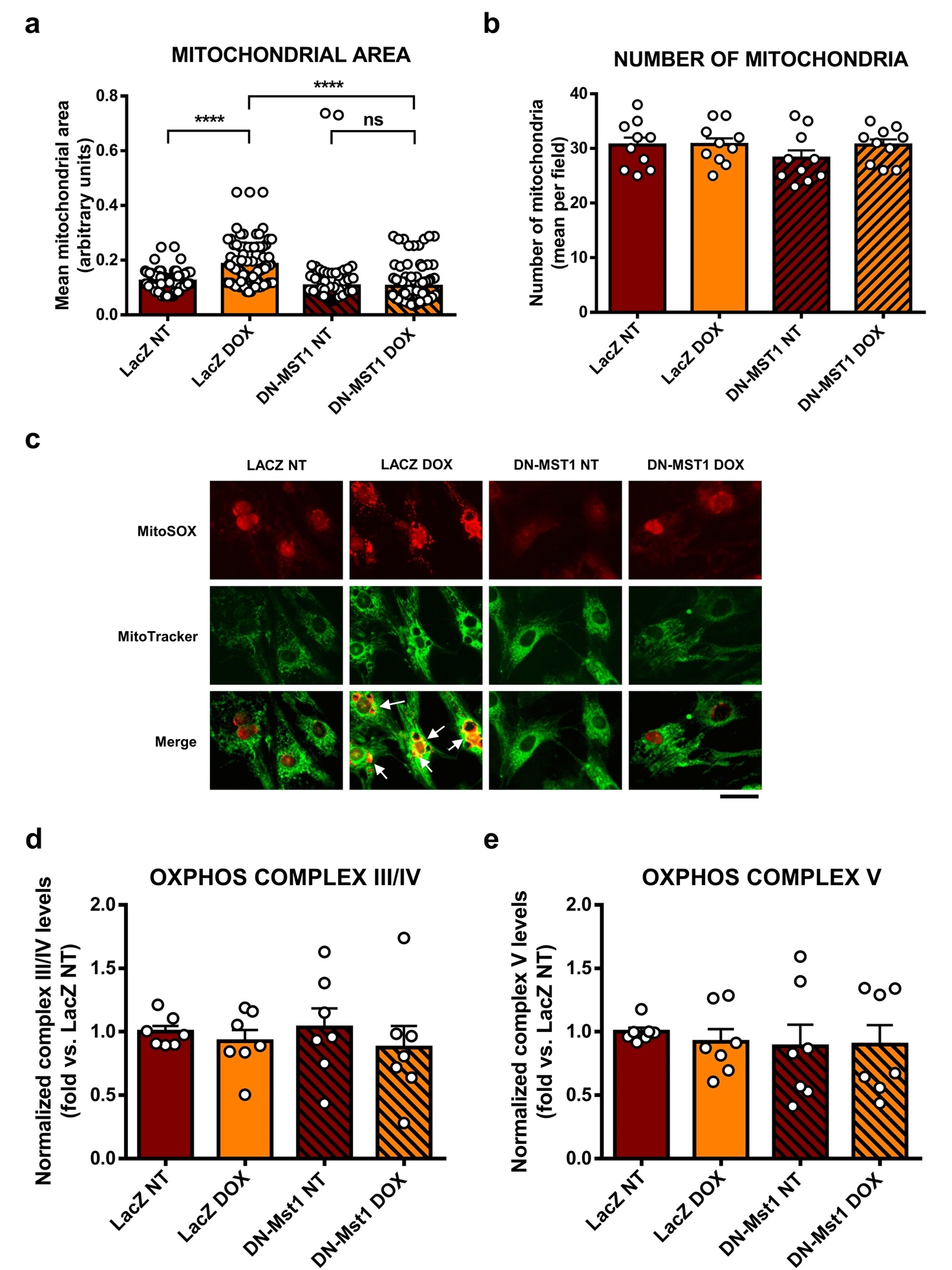
**

**Supplementary Figure 2**


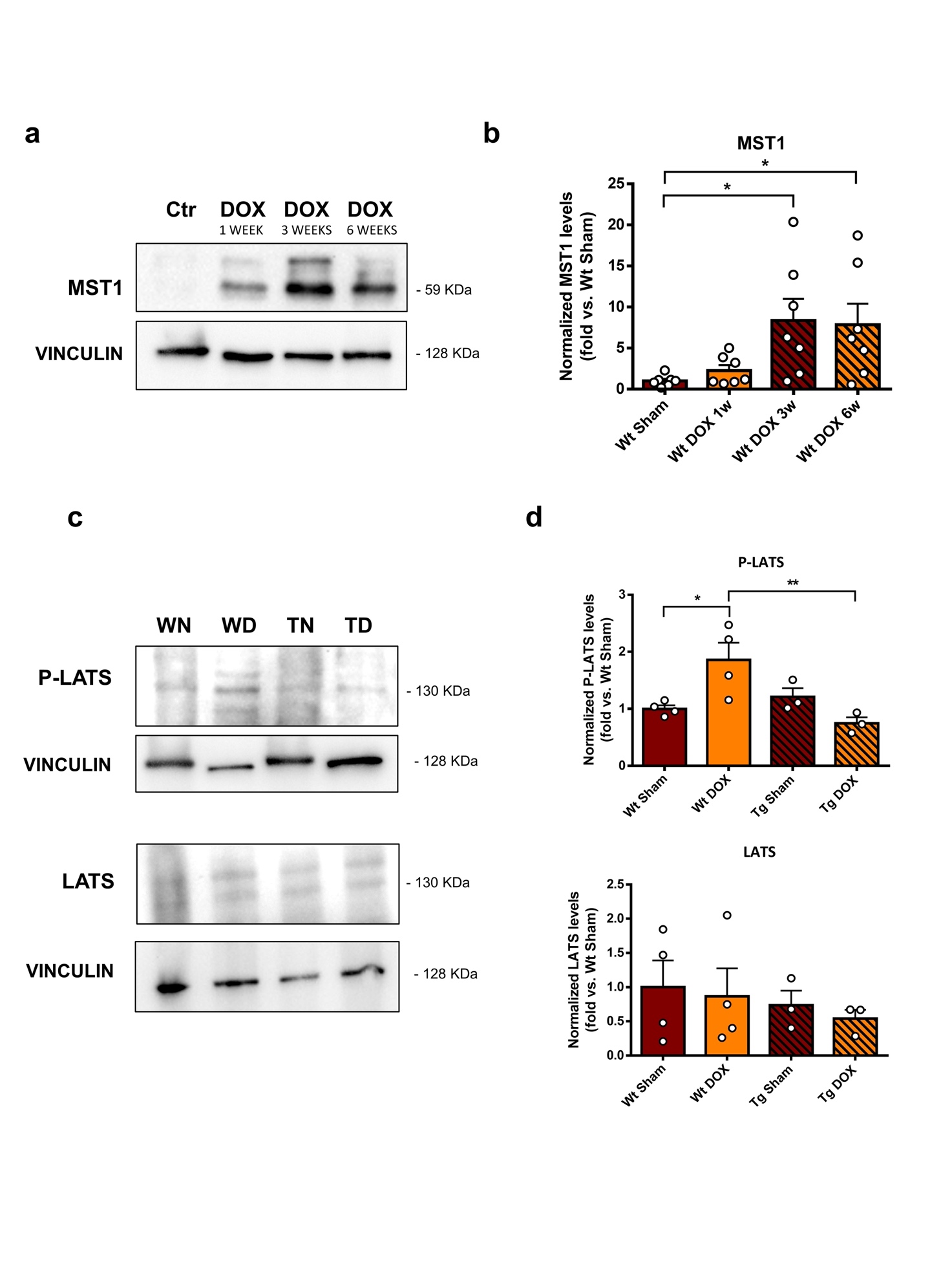


**Supplementary Figure 3**

**
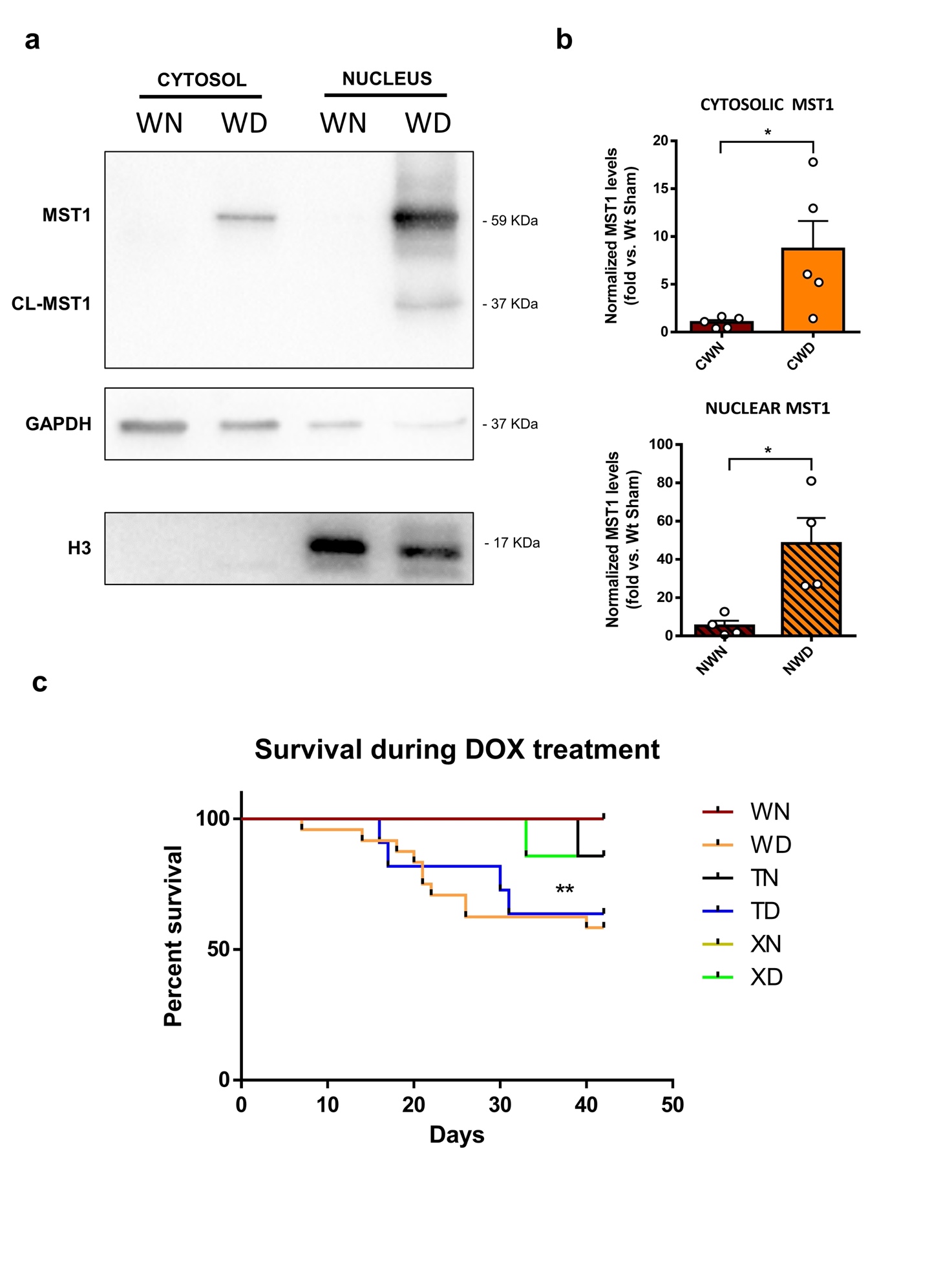
**

**Supplementary Figure 4**

**
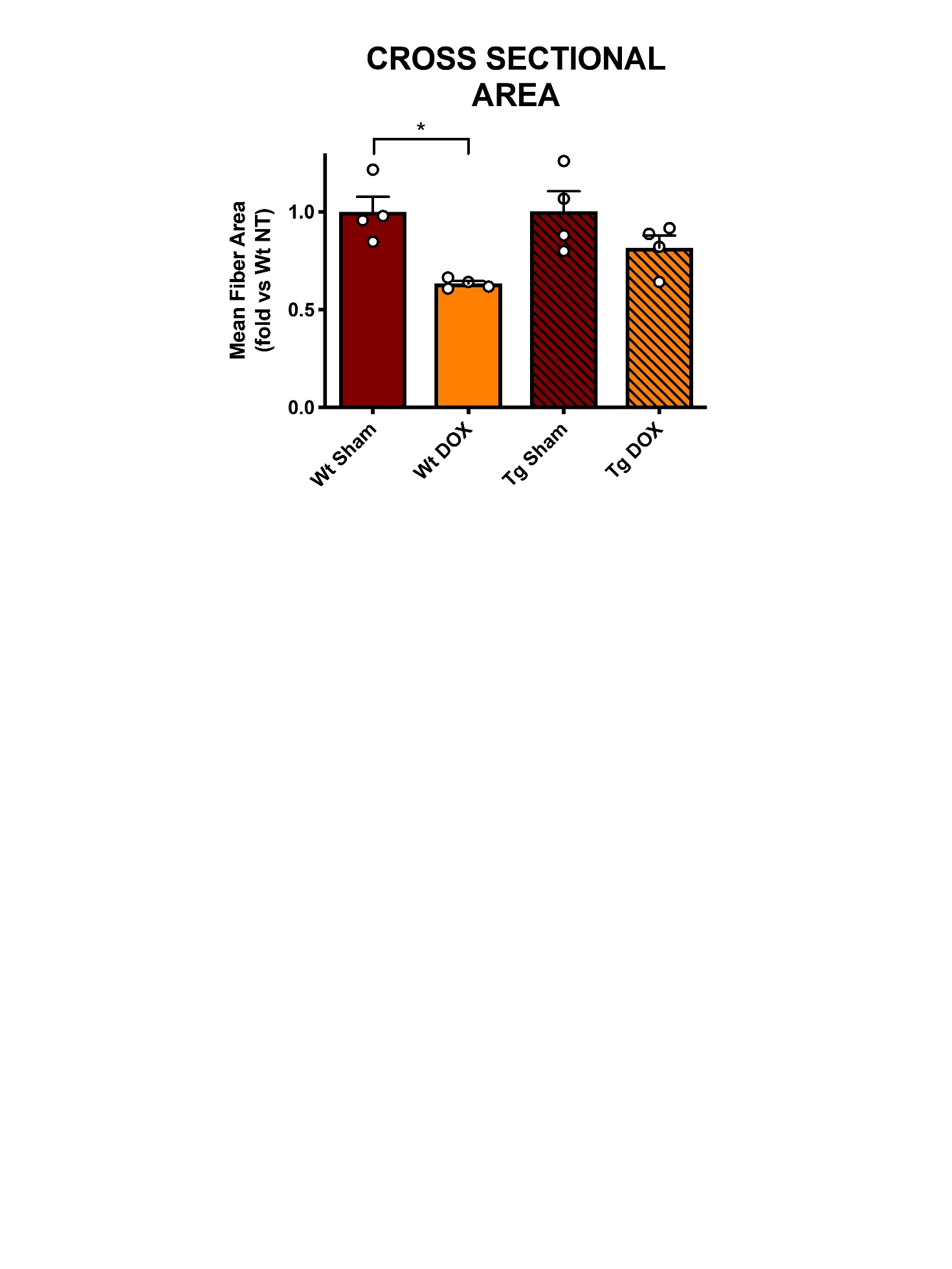
**

**Supplementary Figure 5**

**
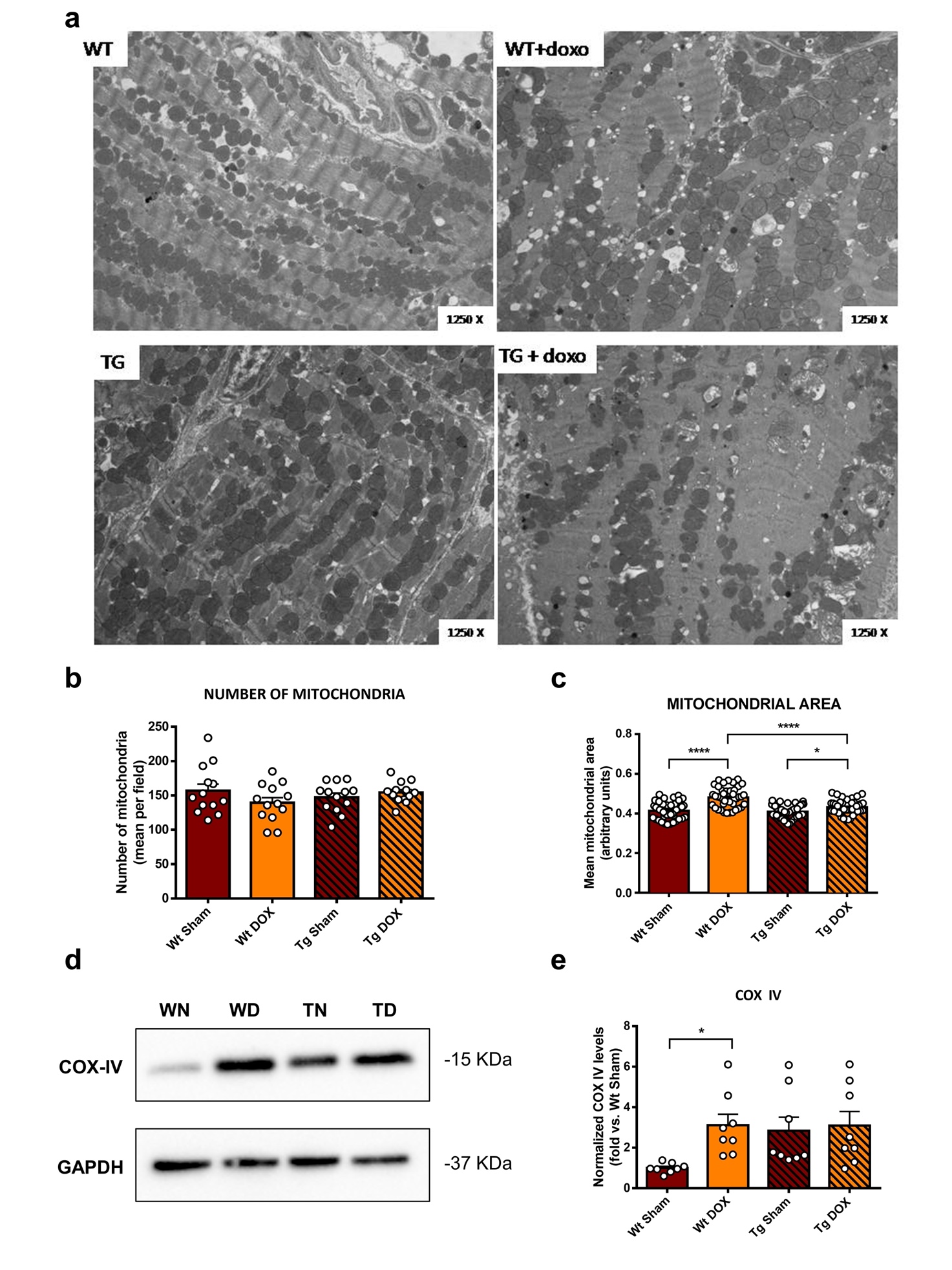
Supplementary Figure 6**

**
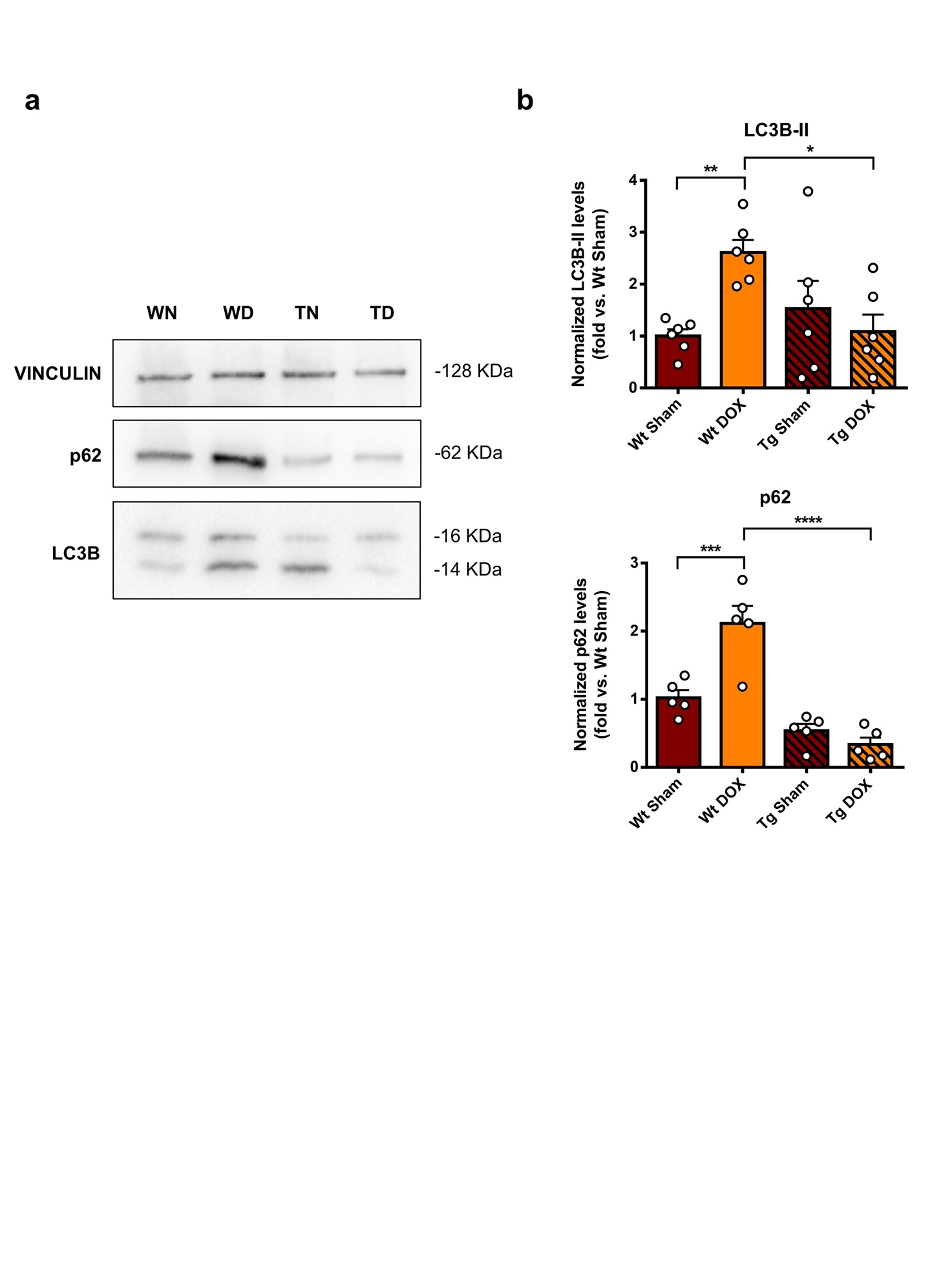
**

**Supplementary Figure 7**

**
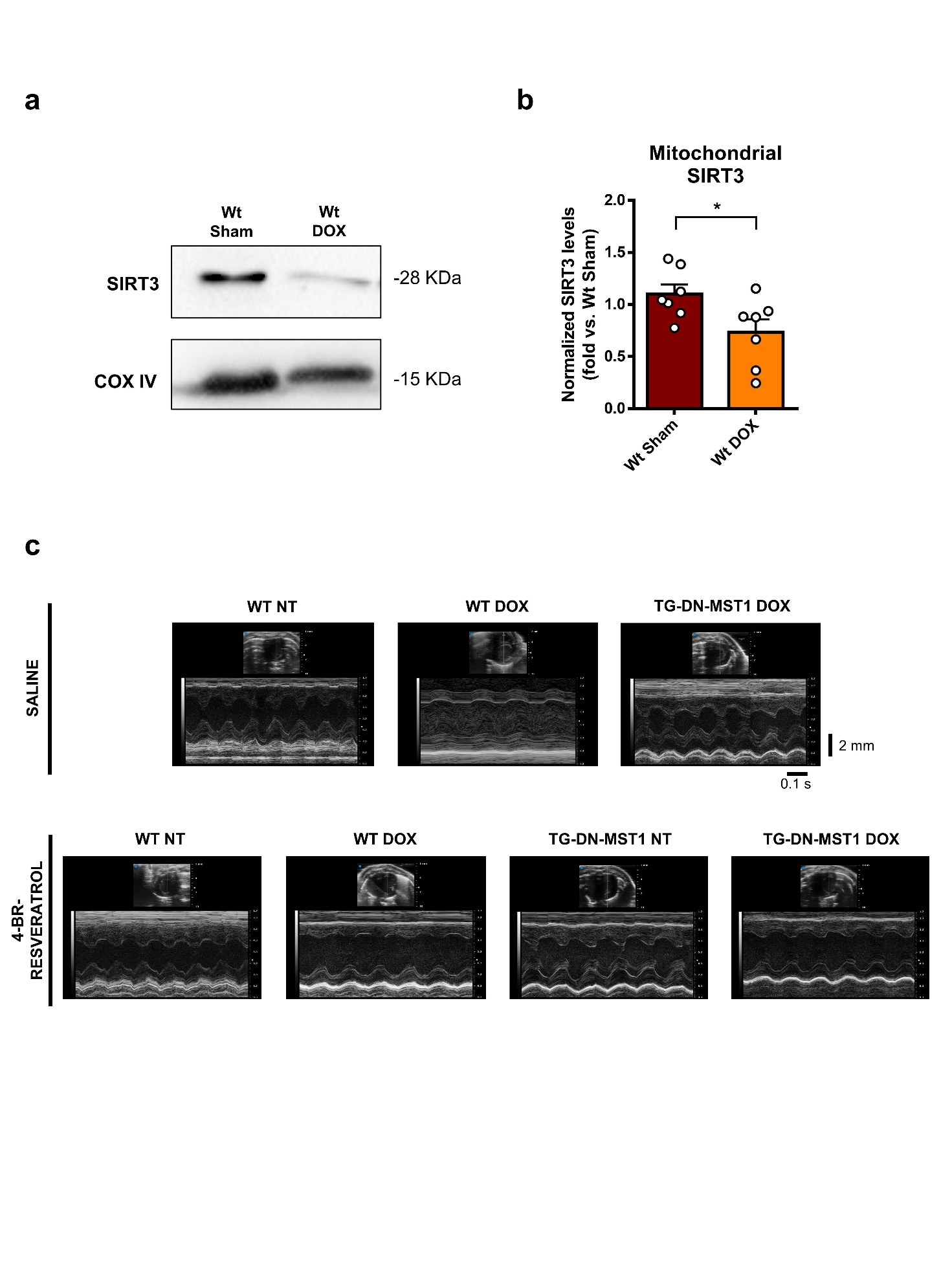
**

**Supplementary Figure 8**

**
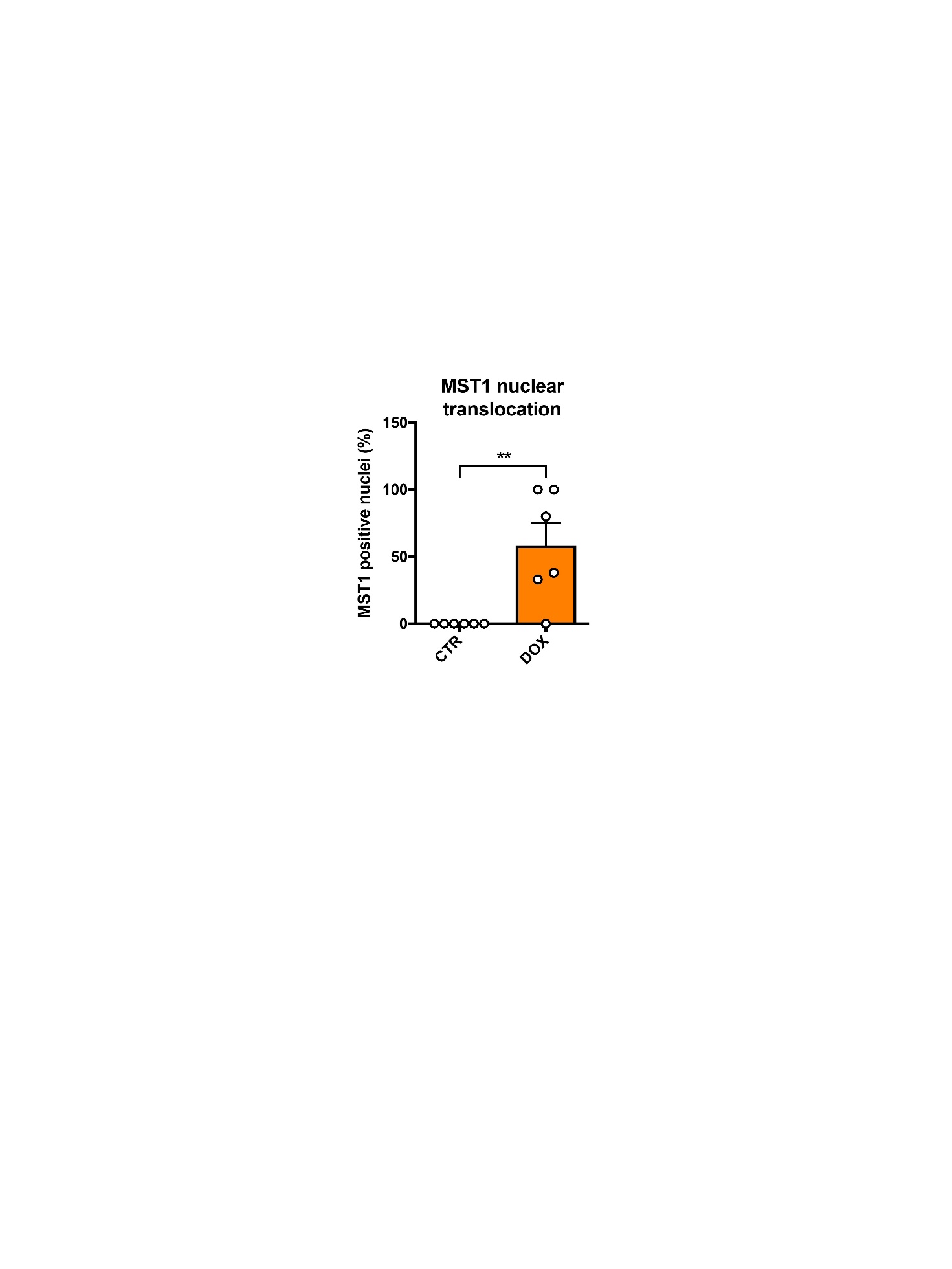
**

**Supplementary Figure 9**

**
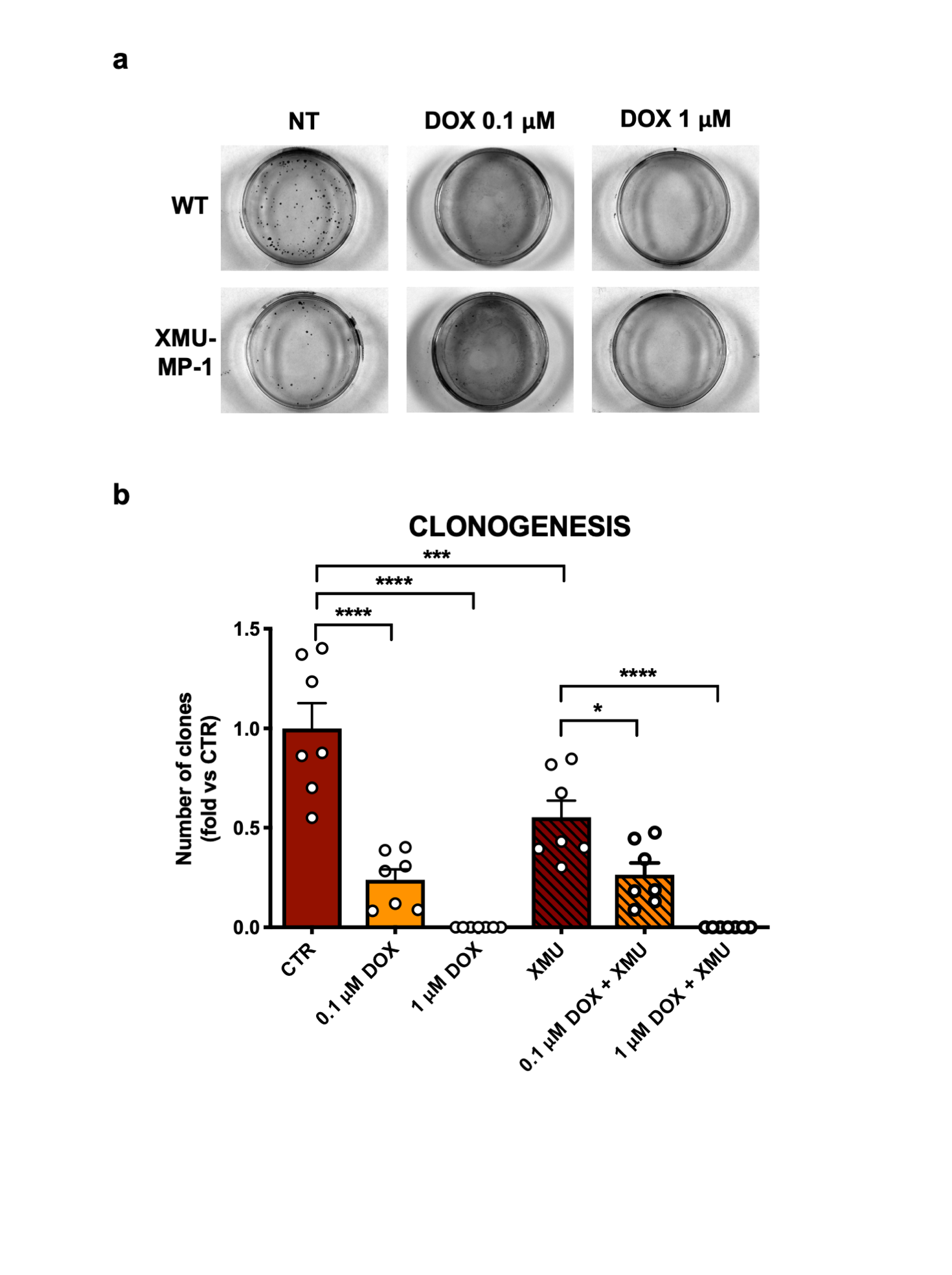
**

**Supplementary Figure 10**
