# Supplementary material for: MST1 mediates doxorubicin-induced cardiomyopathy by SIRT3 downregulation
Source: Cell Mol Life Sci. 2023 Aug 11;80(9):245. doi: 10.1007/s00018-023-04877-7 (PMC10421787; doi:10.1007/s00018-023-04877-7)
Supplement: Supplementary file 3 — Supplementary file3 (DOCX 798 KB) [file 18_2023_4877_MOESM3_ESM.docx]

**SUPPLEMENTARY TABLES**

**Supplementary Table 1**

Table of the echocardiographic measurements and *post-mortem* gravimetric heart analyses after 6 weeks of treatment with doxorubicin 18 mg/kg in c57BL6J and Tg-DN-MST1 mice. Data represent mean ± SEM (*n* = 7-11 independent samples).

**Supplementary Table 2**

Table of the echocardiographic measurements after 6 weeks of treatment with doxorubicin 18 mg/kg and/or XMU-MP-1 3 mg/kg/week. Data represent mean ± SEM (*n* = 8–10 independent samples).


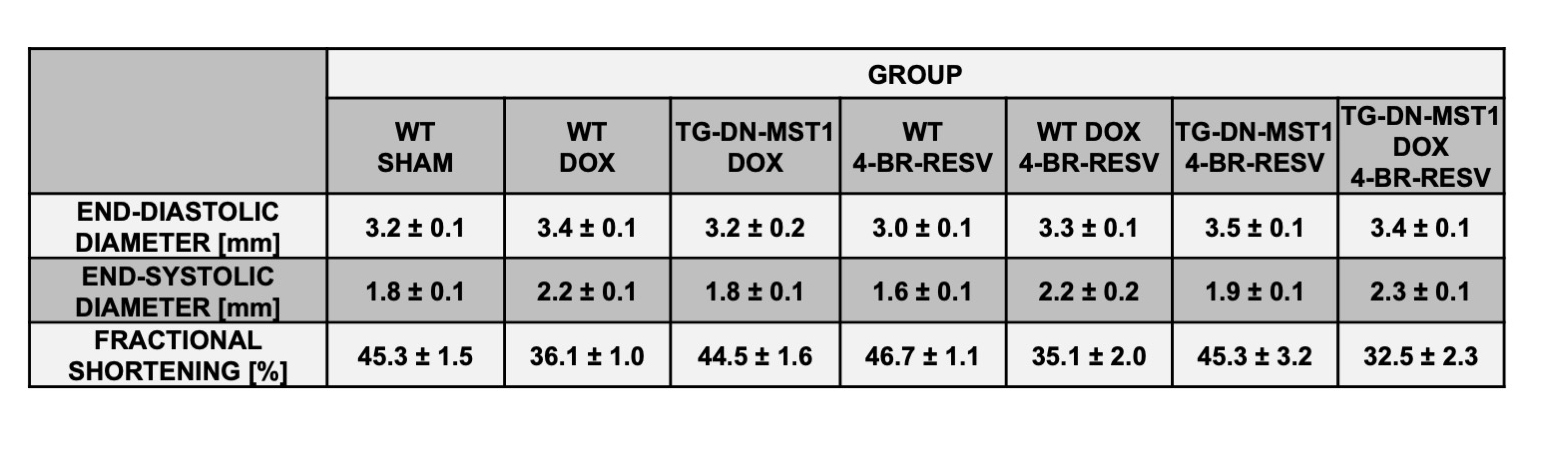


**Supplementary Table 3**

Table of the echocardiographic measurements and *post-mortem* gravimetric heart analyses after 6 weeks of treatment with doxorubicin 18 mg/kg and/or 4’-Br-Resveratrol 30 mg/kg/week. Data represent mean ± SEM (*n* = 4–7 independent samples).


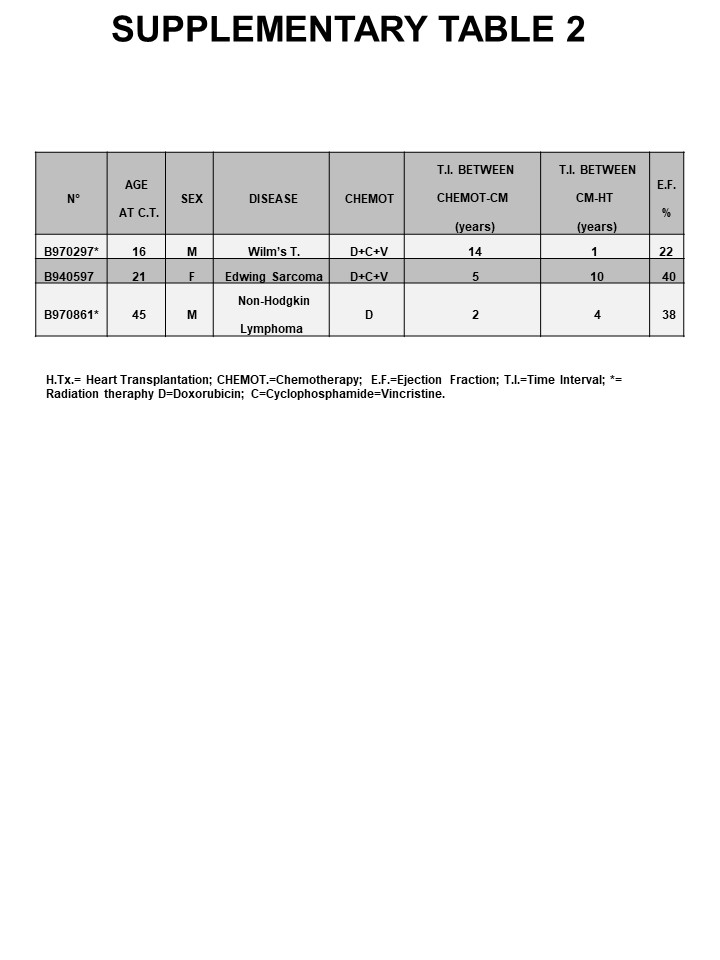


**Supplementary Table 4**

Clinical data table of the donor patients treated with doxorubicin whose specimens were harvested after heart transplantation for the immunofluoremetric study performed. *(H.Tx.= Heart Transplantation; CHEMOT.=Chemotherapy; E.F.=Ejection Fraction; T.I.=Time Interval; *= Radiation therapy D=Doxorubicin; C=Cyclophosphamide=Vincristine.*


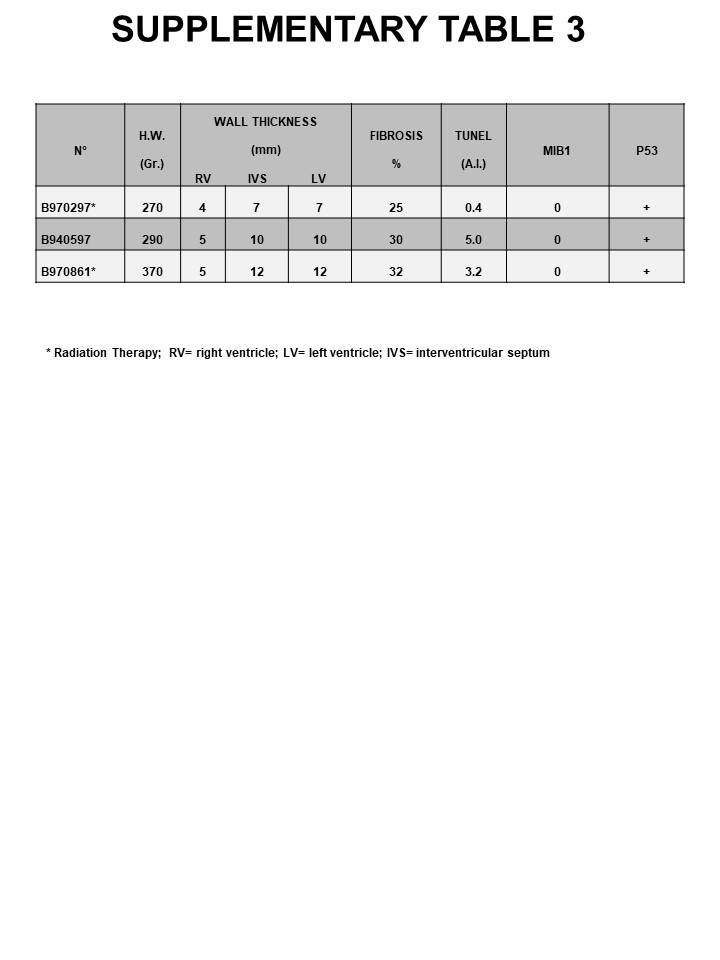


**Supplementary Table 5**

Late-onset cardiomyopathy pathological data table of the donor patients treated with doxorubicin whose specimens were harvested after heart transplantation for the immunofluoremetric study performed. *(* Radiation Therapy; RV= right ventricle; LV= left ventricle; IVS= interventricular septum)*
